# Specialized Metabolites Produced by Phytotopatogen Fungi to Control Weeds and Parasite Plants

**DOI:** 10.3390/microorganisms11040843

**Published:** 2023-03-26

**Authors:** Antonio Evidente

**Affiliations:** Department of Chemical Sciences, Complesso Universitario Monte S. Angelo, University of Naples Federico II, Via Cintia 4, 802126 Naples, Italy; evidente@unina.it

**Keywords:** weeds, parasite plants, pathogen fungi, phytotoxins, bioherbicides

## Abstract

Weeds such as parasite plants are one of the most serious pests that farmers are forced to combat since the development of agriculture using different methods including mechanic and agronomy strategies. These pests have generated significant losses of agrarian and herding production, constituting a serious impediment for agricultural activities in reforestation practices and in important infrastructures. All these serious problems have induced the expansive and massive use of synthetic herbicides, which represents one of the main cause of environmental pollution, as well as serious risks for human and animal health. An alternative environmental friendly control method could be the use of bioherbicides based on suitably bioformulated natural products, of which the main ones are fungal phytotoxins. This review covers the literature from 1980 to the present (2022) and concerns fungal phytotoxins with potential herbicidal activity in order to obtain their efficacy as bioherbicides for practical application in agriculture. Furthermore, some bioherbicides based on microbial toxic metabolites are commercially available, and their application in field, mode of action and future perspectives are also discussed.

## 1. Introduction

Weeds, including parasite plants, are among the most dangerous biotic stresses that attack agrarian, forest and ornamental plants. They cause crops marked losses in agrarian production [1]. The request to satisfy human need had become an urgent problem with the increase over time of world population, which could reach almost 10 billion by 2050 [2,3]. In addition, the rapid spread of weeds worldwide reduces the areas available for grazing, and these plants also are poisonous and harmful to grazing animals [4] and the forest and park heritage [5,6,7]. In addition, weeds create serious problems for important infrastructures [8,9]. Another severe consequence due to weed diffusion in the forest landscape is the drastic reduction in precious wood production [10,11].

The growth of weeds is in big competition with agrarian crops and forest plants by the subtraction of water, nutrients and light, and represents serious obstacles for agronomic activities [12,13]. The main control method used in the last five to six decades has been the massive and extensive use of synthetic herbicides with heavy effects on crop safety, risk for human and animal health and the increasing of environmental pollution, which noteworthily contributes to climate changes. These negative effects have increased with the rise of parallel weed resistance and the consequent increase in treatment repetitions [12,14,15]. These problems prompt the finding of alternative and ecofriendly strategies to control weeds, in particular those based on the use of natural compounds, which are biodegradable and not toxic. The fungal phytotoxins alone or in integrated strategy have been extensively studied as one of the more efficacious methods to combat weeds [16,17,18].

Fungal phytotoxins are secondary metabolites that play an important role in the induction of disease symptoms in agrarian and forest plants and weeds, and belong to different classes of naturally occurring compounds [19,20,21,22,23,24,25,26].

Considering the several publications on this topic and the importance of the arguments treated, some reviews were previously reported. The first two referred to a few fungal phytotoxins with potential herbicide activity [27,28], some others deal with the use of fungal and their suitable formulates as mycoherbicides [29] and some discuss this topic together with the use of allelochemicals [30]. A reviewer reports as potential mycoherbicides only the *Colletotrichum* species [31], while another treats toxicological risks linked to the use of microorganisms as bio-herbicidal and the environmental damage due to their repeated augmentative applications [32]. Some other reviews, which are referred to in the successive decades (2000–2020), describe the fungal phytotoxins with potential herbicide activity as a new tool for weed control, also detailing their production, purification and chemical characterization procedures [24,33,34]. Some others only describe the fungal phytotoxins proposed as potential bioherbicide for the control of a specific weed as *Cirsium arvense*, *Sochus arevensis* [13] and *Chenopodium album* [35]. A review describes the secondary volatile and not volatile metabolites biosynthesized by endophytic fungi [36]. Some other reviews report about the possibility to use fungal toxins to biological control parasite plants, in particular *Pelipanche*, *Orobanche*, *Striga* and *Cuscuta* spp., using different strategies. The first method is based on the use of fungal toxins and natural compounds from different sources able to inhibit the seed germination or to induce the so-called “frenching disease” [37], while the other caused the so-called “suicidal germination” induced by both fungal and vegetable metabolites [15]. A review reports about polyphenols produced by fungal pathogen of crops and forest plants with potential herbicidal and fungicides activities [38]. 

Considering the several publications on this topic and the importance of the arguments treated, some reviews were previously reported. The first two referred to a few fungal phytotoxins with potential herbicide activity [27,28], some others deal with the use of fungal and their suitable formulates as mycoherbicides [29] and some discuss this topic together with the use of allelochemicals [30]. A reviewer reports as potential mycoherbicides only the *Colletotrichum* species [31], while another treats toxicological risks linked to the use of microorganisms as bio-herbicidal and the environmental damage due to their repeated augmentative applications [32]. Some other reviews, which are referred to in the successive decades (2000–2020), describe the fungal phytotoxins with potential herbicide activity as a new tool for weed control, also detailing their production, purification and chemical characterization procedures [24,33,34]. Some others only describe the fungal phytotoxins proposed as potential bioherbicide for the control of a specific weed as *Cirsium arvense*, *Sochus arevensis* [13] and *Chenopodium album* [35]. A review describes the secondary volatile and not volatile metabolites biosynthesized by endophytic fungi [36]. Some other reviews report about the possibility to use fungal toxins to biological control parasite plants, in particular *Pelipanche*, *Orobanche*, *Striga* and *Cuscuta* spp., using different strategies. The first method is based on the use of fungal toxins and natural compounds from different sources able to inhibit the seed germination or to induce the so-called “frenching disease” [37], while the other caused the so-called “suicidal germination” induced by both fungal and vegetable metabolites [15]. A review reports about polyphenols produced by fungal pathogen of crops and forest plants with potential herbicidal and fungicides activities [38]. 

Considering the several publications on this topic and the importance of the arguments treated, some reviews were previously reported. The first two referred to a few fungal phytotoxins with potential herbicide activity [27,28], some others deal with the use of fungal and their suitable formulates as mycoherbicides [29] and some discuss this topic together with the use of allelochemicals [30]. A reviewer reports as potential mycoherbicides only the *Colletotrichum* species [31], while another treats toxicological risks linked to the use of microorganisms as bio-herbicidal and the environmental damage due to their repeated augmentative applications [32]. Some other reviews, which are referred to in the successive decades (2000–2020), describe the fungal phytotoxins with potential herbicide activity as a new tool for weed control, also detailing their production, purification and chemical characterization procedures [24,33,34]. Some others only describe the fungal phytotoxins proposed as potential bioherbicide for the control of a specific weed as *Cirsium arvense*, *Sochus arevensis* [13] and *Chenopodium album* [35]. A review describes the secondary volatile and not volatile metabolites biosynthesized by endophytic fungi [36]. Some other reviews report about the possibility to use fungal toxins to biological control parasite plants, in particular *Pelipanche*, *Orobanche*, *Striga* and *Cuscuta* spp., using different strategies. The first method is based on the use of fungal toxins and natural compounds from different sources able to inhibit the seed germination or to induce the so-called “frenching disease” [37], while the other caused the so-called “suicidal germination” induced by both fungal and vegetable metabolites [15]. A review reports about polyphenols produced by fungal pathogen of crops and forest plants with potential herbicidal and fungicides activities [38]. 

Thus, the present review is the first one focused on only fungal metabolites with herbicidal activity. The results discussed in the two different sections were obtained from Sci-Finder research from 1980 to the present (2022) and chronologically reported. Section 3 describes fungal phytotoxins for the control of weeds, and the fourth section discusses fungal toxins regarding the control of parasite plants. This review is focused on fungal phytotoxins with potential herbicidal activity so as to obtain their efficacy as bioherbicides for practical application in agriculture and other fields. Furthermore, the bioherbicide based on microbial toxic metabolites commercially available, and their application in field, their mode of action and future perspectives are also discussed.

## 2. Recent Developments for the Purification and Identification of Natural Compounds

Innovative technologies have played an important role in the chemistry of natural compounds. As regards the compound purification from a complex mixture, HPLC (high-performance liquid chromatography) has assumed a crucial role in addition to the traditional chromatographic methods by column and TLC. HPLC is routinely used in the separation of natural compounds and the development of its hyphenated techniques, as LC/UV-photodiode array (LC/UV-DAD), LC/mass spectrometry (LC/MS) and LC/nuclear magnetic resonance (LC/NMR) provide very efficacious tools to reach the fundamental goal of having a pure natural compound to submit to its identification. Biological samples managing the preparation of the sample to apply to the columns is a very important step. In fact, the sample may contain complex macromolecules and contaminants which could damage the column and are frequently expensive. Thus, these contaminants should be preliminary removed [39]. The chemical composition of a given mixture of natural compounds is generally analyzed conjointly by a gas chromatography-flame ionization detector (CG-FID) and GC-mass spectrometry (MS) techniques for quantitative and qualitative purposes. The identification of compounds is performed by comparison of the experimental results with those reported in the electron ionization mass spectrometry (EI-MS) spectra library. However, in many cases (i.e., less than 90% of agreement), the identification is not unambiguous and further investigation are necessary. To have the confirmation of the structure of the compound analysed, the NMR methodologies (1D ^1^H, ^13^C and/or two-dimensional (2D) NMR) must be used. NMR is the most powerful tool for the structural determination of organic compounds which frequently are completely novel and often possess unusual or unprecedent reported structural features. However, when two or more compounds with similar structure are present in the same sample, NMR spectra could contain signal systems which are difficult to assign (e.g., low signal intensity and/or significant chemical shift overlapping). In this context, the joint use of electrospray ionization mass spectrometry (ESI–MS) could be an efficient alternative method. In fact, ESI does not induce the molecular structure changing, and the spectra recorded are substantially devoid of fragment ions. However, significant structural data could only be provided by tandem mass spectrometry experiments (MS/MS), which permit the post-ionization process into a collision cell, and allow controlled ion dissociation [40,41].

In addition, in the last decade, ion mobility mass spectrometry (IM-MS) has been developed that more easily allow the unambiguous identification of an organic compound. In IM-MS experiments, using a buffer gas and applying a weak electric field, two ions that have the same mass-to-ratio pass through the drift cell and are differentially decelerated according to their active surface. This process is called collision cross section (CCS). Thus, the IM-MS method could be very suitable to analysing isomer mixtures [42].

## 3. Fungal Phytotoxins to Biocontrol Weeds

This section chronologically describes, except for the cases of treating the same argument, the source, structure and biological activity of the fungal metabolites which showed potential herbicidal activity to biocontrol weedy plants, and in some cases, other interesting biological activities.

**Figure 1:**
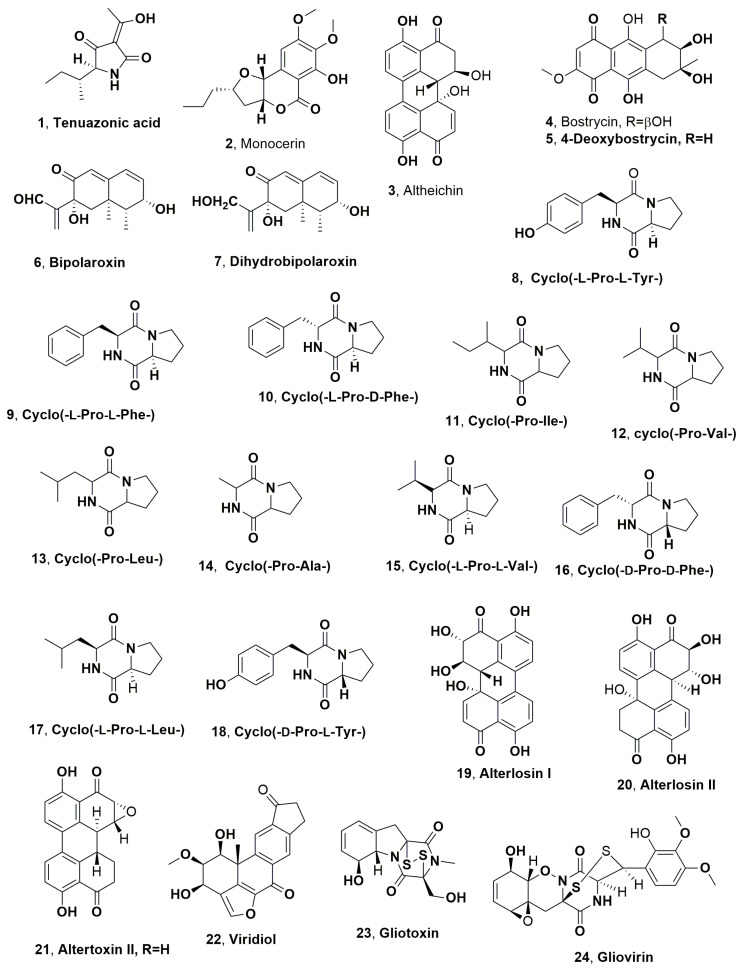
Metabolites produced by *Alternaria alternata* (**1**, **8**–**14**, **19**–**21**), *Exserohilum turcicum* (**2**), *Alternaria eichorniae* (**3**–**5**), *Bipolaris cynodontis* (**6** and **7**), *Lysobacter capsici* (**15**–**18**) and *Gliocladium virens* (**22**–**24**).

Two phytopathogen fungi were isolated from infected leaves of johnsongrass (*Sorghum halepense* L.), which was collected in Israel. Johnsongrass is one of the most dangerous weeds, inducing severe losses to 30 crops in 53 countries [43]. One of its pathogens was identfied as *Alternaria alternata*, which produced the known phytotoxic tenuazonic acid (**1**, Figure 1) [44]. The other fungus was identified as *Exserohilum turcicum* (also reported as *Drechslera turcica*), which is a known pathogen of sorghum and zea leaves causing elongated pale spots, often with a dark margin [45]. Compound **1** was firstly isolated from the culture filtrates of *Alternaria longipes*, which is the responsible agent of the tobacco brown spot disease. When a few drops (20 μL each) of aqueous solution containing different amount of compound **1** were applied to the tobacco leaf at 25–30 °C, the toxin induced necrotic spots surrounded by halos, which appeared within 60 h after inoculation [46]. *E. turcicum* also produced another phytotoxic compound, which was named monocerin (**2**, Figure 1). The latter compound (**2**) showed phytotoxic activity when assayed on cuttings of Canada thistle (*Cirsium arvense*), inducing a confluent necrotic spot 7 mm in diameter within 16 h after treatment. The necrotic area expanded with the increasing of time. When tested in the same condition on tomato (*Lycopersicon esculentum*) cuttings, compound **2** caused the wilting of leaves, while the tissues became flaccid within 16 h after treatment. Finally, when assayed against johnsongrass at 1 mg/mL, monocerin compound **2** almost completely inhibited root elongation of pre-germinated seeds, and caused root necrosis. When it was tested at the lowest concentration (33 ppm), it induced approximately 50% reduction in root elongation after 96 h, as compared with controls. Tested at same concentration and time of incubation on seedling shoot growth, compound **2** caused a similar decrease [47].

Altheichin (**3**, Figure 1), which is a doubly hydrated form of 4,9-dihydroxy perylene-3,10-quinone, was isolated from *Alternaria eichorniae*, a pathogen of water hyacinth, which is an economically important aquatic weed. Water hyacinth (*Eichornia crassipes*) is a perennial, herbaceous and aquatic plant native to the Amazon basin. It is one of the most dangerous weeds predominantly diffused in the tropics and subtropics world regions. It generated heavy crops losses for rice and represents a great problem for navigable waterways, irrigation canals and drainage ditches. Alteichin (**3**), tested by leaf puncture on the host plant at concentrations of 1, 5 and 10 μg per 10 μL droplets, produced a necrotic fleck within 12 h, which grew to 3–4 mm in diameter after 5 days. Compound **3**, assayed at similar concentrations, also caused necrotic lesions on tomato, Canada thistle, wheat, sunflower and barley leaves [48]. Alteichin (**3**) directly affected some sites in the plant cell, similar to the action of cercosporin, which causes structural changes in plant membranes [48].

Previously, two pigments from the same fungus were isolated in the ratio of 4:1 and identified as the two anthraquinone derivatives, which were named bostrycin and 4-deoxybostrycin (**4** and **5**, Figure 1). Compounds **4** and **5**, when tested on the leaves of host plant and those of some agrarian and weedy plants, showed phytotoxicity, with necrosis ranging <1 to 3 mm when assayed at 250 μg/μL. These symptoms were comparable to those induced from the fungus producer culture filtrates, but at four-fold concentrated. Furthermore, the two toxins did not show toxicity when tested on the shoots of the aquatic plant hydrilla (*Hydrilla verticillata*) [49].

Bipolaroxin and dihydrobipolaroxin (**6** and **7**, Figure 1), which are two sesquiterpenoids belonging to the subgroup of eremophilanes, were isolated from *Bipolaris cynodontis* pathogen of Bermuda grass (*Cynodon dactylon* L.), which is recognised in 80 countries as a weed problem in at least 40 different crops, and is also known as one of the causers of “hay fever”. Bipolaroxin (**6**) was tested at a concentration range 0.038–3.8 mM against dicots such as *Zea mays* (corn), *Helianthus annus* (sunflower), *Saccharam s*pp. (sugarcane) *Eleucine indica* (goosegrass), *Festuca* sp. (fescue), (wild oats), *Amaranthus arverse* (pigweed) and the two hosts *Cynodon dactylon* (Bermuda grass) and *S halepense* (Johnson grass), showing selective phytotoxic activity against the two host weeds. Furthermore, when tested at 0.38 mM, it did not induce toxicity on agrarian crops such as wheat (*Triticum aestivum*), barley (*Hordeum vulgare*), cotton (*Gossypium hirsutum*) and corn (*Zea mays*). Its dihydro analogue **7** did not cause phytotoxicity when assayed in the same conditions against Bermuda grass, goosegrass, wheat or barley, showing that the C12 aldehyde is a structural feature essential for activity [50].

Cyclo(-L-Pro-L-Tyr-) (**8**, Figure 1) was produced together with six diketopiperazines from *A. alternata*, which is the causal agent of black leaf blight of spotted knapweed, (*Centaurea maculosa*) [51]. Since the 1900s, the weed has infested rangelands of the northwestern United States and southwestern Canada, causing ca. 70% losses in forage production [52]. Furthermore, *C. maculasa* rapidly spread in the absence natural predators, and it is efficacious enough to compete with native grasses. The other six diketopiperazine were identified as cyclo(-L-Pro-L-Phe-), cyclo(-L-Pro-D-Phe-), cyclo(-Pro-Ile-), cyclo(-Pro-Val-), cyclo(-Pro-Leu-) and cyclo(-Pro-Ala-) (**9**–**14**, Figure 1). The relative and absolute configuration of the last four dicyclopeptides was not determined at that time. All the diketopiperazines (**8**–**14**) were tested on knapweed leaf puncture, and hypocotyls and cyclo(-L-Pro-L-Tyr-) caused lesions at concentrations ranging 10^−3^ to 10^−5^ M. Compounds **9** and **10** and showed different phytotoxicity, as the first (**9**) showed toxicity at 10^−4^ M and the other (**10**) inactivity even at 10^−3^ M. The other compounds, **11**, **12**, **13** and **14,** were not toxic against knapweed at any of the test concentrations. Similar results were observed when the test was repeated with hypocotyl tissue. The host specificity of cyclo(-L-Pro-L-Tyr-) (**8**) was determined using leaf puncture assay and with a wide variety of plants including both monocots and dicots, testing the toxin at a concentration range of 10^−3^–10^−5^. Among the 19 tested plants, which were infected from different *A. alternata* form, there are different agrarian crops and weeds, and compound **8** induced consistent necrotic lesions only in knapweed leaves [51]. Later cyclo(-L-Pro-L-Tyr-) (**8**) was also produced by *Lysobacter capsici* AZ78 and showed antifungal activity against sporangia of *Phytophthora infestans* and *Plasmopara viticola* [53]. These two pathogens are, respectively, the causal agent of the late blight of potato (*Solanum tuberosum*) and tomato (*Solanum lycopersicum*) [54], as well as the the downy mildew of grapevine (*Vitis vinifera*) [55]. The application of *L. capsici* AZ78 culture filtrates appeared to be a convenient and ecofriendly alternative method to control the two diseases with respect to the traditional methods based on copper products, which possess a negative environmental impact and risks for human and animal health. A similar activity to protect the tomato plant from *P. infestans* was showed from the bacterial culture filtrates [53]. Cyclo(-L-Pro-L-Tyr-) (**8**) and its four hemisynthetic derivatives prepared from compound **8** were used in a structure–activity relationship (SAR) study aimed to obtain a safe and environmentally friendly specific bioherbicide. The derivatives of compound **8** were obtained by the esterification of the phenol hydroxy group with the acetic anhydride, *p*-bromobenzoyl chloride, 5-azidopentanoic and 2-naphthoic acids [56]. The dicyclopeptide **8** and its four derivatives were tested for their antifungal activity against *P. infestans*. The results obtained showed that the antifungal activity increased with the polarity of the compounds. In fact, the acetyl and the naphthoyl ester of **8**, which by cromatocgraphic profile were more polar compared to compound **8**, and its *p*-bromobenzoyl and 5-azidopentanoyl esters showed increased activity [56]. The polarity of the derivatives could facilitate their crossing of the cell membrane, and when inside they could be hydrolyzed in the active phenolic form, a process that frequently occurs among the natural compound esters at physiological pH [57]. In addition, the very strong fungicidal activity of the 5-azidopenatoyl ester of compound **8** could also be increased by its reactivity. Indeed, in losing nitrogen, the esters could be converted in the corresponding very reactive nitrene, which could link with lone electron pair-bearing groups of the receptor binding sites [58,59]. 

*L. capsici* showed how to produce the other 2,5-diketopiperazines which showed toxicity against *P. infestans* and *P. viticola*. These were identified as cyclo(-L-Pro-L-Val-), cyclo(-D-Pro-D-Phe-), cyclo(-L-Pro-L-Leu-) and cyclo(-D-Pro-L-Tyr-) (**15**–**18**, Figure 1) [60]. When tested against the phytopathogen Gram-positive bacterium *Rhodococcus fascians*, only compounds **8** and **15** showed antibiotic activity, reducing the bacterial viability by 16 and 5%, respectively, compared to the untreated control, and similar to that of chloramphenicol, which was used as a positive control [60]. Recently, cyclic dipeptides (cyclo-L-Pro-L-Tyr-), cyclo(-L-Pro-L-Val-), cyclo(-L-Pro-L-Leu-) and cyclo(-D-Pro-L-Tyr-) (**8**, **15**, **17** and **18**), together with the bacterial lipodepsipeptides such as tolaasins I, II, A, D and E, and WLIP together with hexacetyl- and tetrahydro-tolaasin I and WLIP methyl ester, were tested against several pathogenic bacteria and fungi. These latter included the *Burkholderia caryophylli* (syn. *Pseudomonas caryophylli*) responsible for bacterial wilt of carnation, causing serious losses in carnation production [61] and *Pseudomonas syringae* pv. *panici*, which induces diseases in different plants including rice, lilac, millet and pearl millet [62] as well as *Pseudomonas syringae* pv. *tabaci*, responsible for brown spots on tobacco, a disease named wildfire which causes severe economic consequences [63]. *Pseudomonas syringae* pv. *siringae*, which is the most polyphagous bacterium in the *P. syringae* complex affecting woody and herbaceous host plants [64], was also used together with *Pseudomonas syringae* pv. *japonica*, which is the causal agent of the black node disease of barley (*Hordeum vulgare* L.) and wheat (*Triticum aestiuum* L.) [65]. *Bacillus subtilis*, *Bacillus megaterium* and *Escherichia coli* were used as common laboratory strains. *Colletotrichum truncatum*, which is responsible for the dangerous soybean anthracnose in Argentina causing significant yield losses [66], is the only fungus used. The results obtained showed that the antibacterial and antifungal activity of lipodepsipeptides in the inhibition of microbial growth was 56–60 times higher than that of dicylopeptides. Among the lipodepsipeptides, the nonapeptides such as WLIP exhibited weaker fungicide activity against *C. truncatum*. The presence of some amino acid residues of the lactone ring of lipodepsipetides with a longer peptide side chain is an important feature in increasing activity, while the derivatization of the amino acid residues of both the macrocyclic lactone ring and linear peptide side chain weakly affects inhibitory activity [67].

Successively, *A. alternata*, which is described above as the causal agent of black leaf blight of spotted knapweed (*Centaurea maculosa*) [68], showed production alongside the diketopiperazine (**8**–**14**) and also other phytotoxins belonging to tetramic acids and perylenequinones families. In fact, tuenazoic acid (**1**), which was described aboce, was isolated from the fungal culture together with four perylenequinones such as alterlosin I and II (**19** and **20**, Figure 1), altertoxin III (**21**, Figure 1) and alteichin (**3**) [68]. The latter compound (**3**) was already reported above as phytotoxic metabolite of *A. eichomiae* [50] and was isolated as a pigment from *A. altemata* together with altertoxin III (**21**), which showed toxicity on lettuce [69] and also appeared to be a mutagenic substance [70]. All four perylenequinones (**19**–**21** and **3**) were tested against knapweed, and alterlosins I and II (**19** and **20**) caused necrotic lesions at test concentrations of 10^−4^ M, with compound **20** inducing larger necrotic lesions compared to the small flecks induced by compound **19**. Compounds **3** and **21** were not toxic to knapweed at any test concentration [68].

Viridiol (**22**, Figure 1) was isolated as the main phytotoxin from the cultures of *Gliocladium virens* when the fungus was grown on rice [71]. *G. virens* produces a number of metabolites with antimicrobial activity such as gliotoxin [72] and gliovirin [73] (**23** and **2**4, Figure 1), which have antifungal and antibacterial activity. When the fungus peat mixture was applied at rates of 8.7% (of total volume) or less, most weeds were reduced >90%, while seedling dry weights also showed marked reduction. Similarly, applications of the same mixture at 4.5% induced the reduction in root and shoot weight of redroot pigweed by 93 and 98%, respectively. Crops were only affected at higher treatment levels. Viridiol production was detected 3 days after the incorporation of the fungus-peat mixture at a rate of 11%, reaching the peak on the fifth and sixth days. The values measured were proximately 25 ug viridiol/100 mL soil, and then a decrease to undetectable levels by the end of 2 weeks occurred. Compound **22** showed phytotoxicity against an annual composite species but was less toxic in monocot control [71].

Successively, viridiol (**22**) was isolated together with some analogues such as 1-deoxyviridiol, nodulisporiviridin M, demethoxyviridiol and hyfraxinic acid, which is a tetrasubstituted octanoic acid from the organic extract of *Hymenoscyphus fraxineus*. The fungus was responsible for ash (*Fraxinus excelsior* L.) dieback in Europe. All the compounds were tested at concentrations of 1.0 and 0.5 mg/mL by a leaf puncture assay on *Celtis australis* L., *Quercus suber* L., *Hedera elix* L., *Juglans regia* L. and *Fraxinus angustifolia* L. Among them, hyfraxinic acid, viridiol and demethoxyviridiol showed strong phytotoxic activity on different Quercus spp. inducing necrotic lesions, while 1-deoxyviridiol and nodulisporiviridin M appeared to be inactive [74].

**Figure 2:**
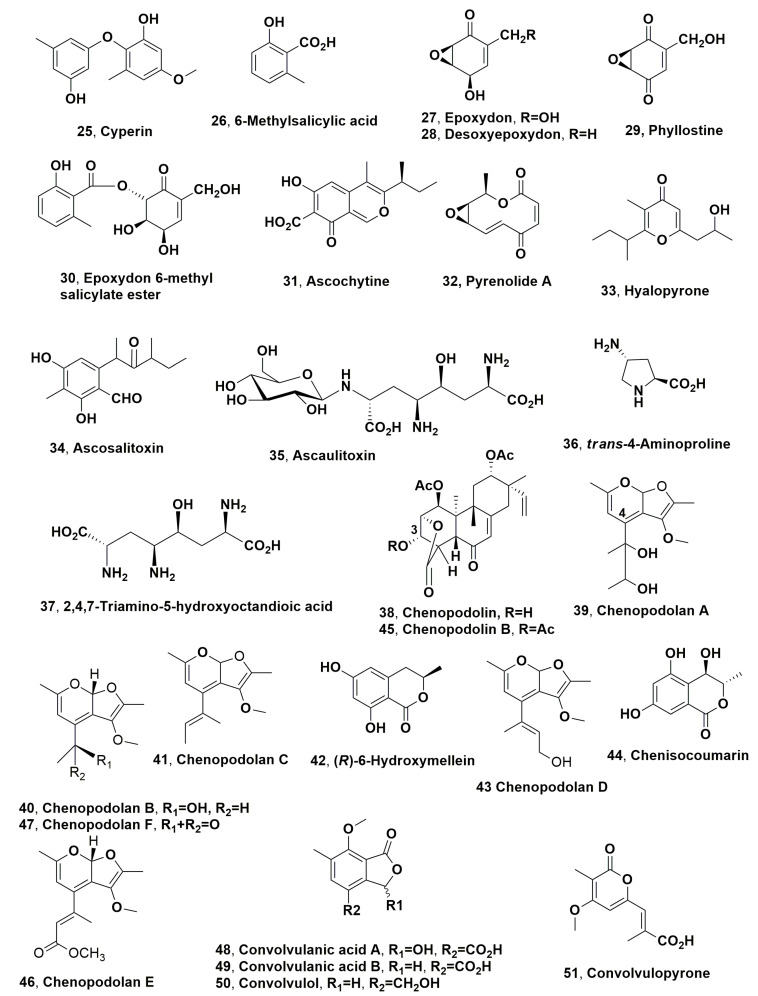
Metabolites produced by *Ascochyta cypericola* (**25**), *Phoma sorghina* (**25**–**30**), *Ascochyta hyalospora* (**31**–**33**), *Ascochyta pisi* (**34**), *Ascochyta caulina* (**35**–**37**), *Phoma chenopodiicola* (**38**–**47**), *Phomopsis convolvulus* (**48**–**51**).

Cyperine (**25**, Figure 2) was produced from *Ascochyta cypericola* isolated from purple nutsedge (*Cyperus rotundus* L.), which is a well-known and ubiquitous weed that is widespread throughout Africa, India, Southern Asia, Australia, South and Central America and the southern United States [75]. *A. cypericola* induces both sunken lesions on the stem, sheath and involucre, as well as reddish-brown striations on the leaves of infected plants. Cyperine (**25**) is a strong phytotoxin with modest host selectivity within the genus *Cyperus*. The host weed (*Cyperus rotundus* L.) showed the greatest sensitivity to the toxin among the other species tested [76].

Cyperin (**25**) was also isolated together with 6-Methylsalicylic acid, epoxydon, desoxyepoxydon, phyllostine and epoxydon 6-methylsalicylate ester (**26**–**30**, Figure 2) from *Phoma sorghina*, which was obtained from leaf spots on pokeweed (*Phytolacca americana* L.) [77]. Pokeweed is widespread throughout southeastern United States in disturbed habitats, and when infected by *P. sorghina*, showed leaves lesions which appeared brownish-black and ranged from 5 to 12 mm in diameter. The fungus is also well known as the causal agent of leaf spots on dicot crop plants and glume blight of rice [78,79,80,81,82,83]. The compounds **25**–**30** induced necrosis on pokeweed leaves and on eight other weed species including sicklepod, Prickly sida, johnsongrass, sorghum, Morning Glory, jimsonweed, lambsquarters and watercress, appearing to be non-specific phytotoxins. Metabolites **25**, **27** and **28** also exhibited strong antimicrobial activity against all bacteria and fungi tested, while compounds **25**, **26** and **27** also inhibited sorghum root growth [77].

Ascochytine, 2 pyrenolide A and hyalopyrone (**31**–**33**, Figure 2) were produced by *Ascochyta hyalospora*, which is a fungus responsible for lambsquarters or fat hen (*Chenopodium album* L.) leaf spot. The compounds **31**–**33** showed phytotoxicity in tree assays using the host plant (lambsquarter) and other different weedy plants such as Prickly sida, sicklepod, Morning Glory, johnsongrass, sorghum, bentgrass, ragweed, watercress and jimsonweed. The strongest phytotoxic activity was showed by assaying ascochytine, the main fungal metabolite (**31**) on johnsongrass and sorghum. Compound **31** and pyrenolide A (**32**) exhibited comparable activity towards the host plant lambsquarters, while hyalopyrone (**33**) was markedly less active. Ascochytine and pyrenolide A also showed a similar activity in causing electrolyte leakage in lambsquarters and in inhibition sorghum *root growth* [84]. Ascocytine (**31**) was also isolated from some strains of *A. pisi* and *A. fabae*, which are well known as pathogens responsible of pea and bean anthracnose when cultivated in liquid medium. *A. pisi* also produced ascosalitoxin, 2,4-dihydroxy-3-methyl-6,1,3di-methyl-2-oxopentyl)benzaldehyde (**34**, Figure 2) when grown autoclave kernels. Compound **34** showed phytotoxic activity on pea and bean leaves and pods, and on tomato seedlings, but had no zootoxicity [85].

Successively, the biocontrol of fat hen (*C. album* L.) was extensively studied with the economic support of the European Project FAIR5-CT97–3525 entitled “Optimizing Biological Control of a Dominant Weed in Major Crop”. *Ascochyta caulina* is a pathogen fungus which specifically infected *Chenopodium album* L., and was suggested as a potential mycoherbicide to control this weed, which is very dangerous and widespread in arable crops throughout Europe [86,87]. Ascaulitoxin, its aglycone (2,4,7-triamino-5-hydroxyoctandioic acid) and *trans*-4-aminoproline (**35**, **37** and **36**, Figure 2) were isolated as phytotoxic metabolites from the fungal culture filtrates of *A. cauilina* [88,89,90]. The relative and absolute configuration of ascaulitoxin and *trans*-4-aminoproline (**35** and **36**) were, respectively, determined. That of toxin **35** was determined by studies on *J*-based NMR configurational analysis of a nitrogen-substituted system [91], while that of compound **36** was assigned through its enantioselective synthesis [89]. A HPLC method was developed for the qualitative and quantitative analysis of the three phytotoxins using an anion exchange chromatography column and a pulsed amperometric detection [90]. A simple, convenient and ecofriendly method for large-scale preparation of the mixture of the three toxins by cation exchange chromatography was developed, affording an amount of phytotoxin to further characterize their herbicidal potential in green-house and field [92]. Ascaulitoxin and *trans*-4-aminoproline (**35** and **36**) were assayed to evaluate their phytoxicity, using 30 μg per droplet on punctured leaves of different plants including the host, other weeds and cultivated species. Compound **35** induced necrotic spots surrounded by chlorosis on *C. album* leaves and on other weeds (common sowthistle, annual fleabane, noogoora burr, tree of heaven), and on cultivated plants (pea). When assayed on tomato and redroot pigweed, it caused necrosis of reduced size. Compound **36**, using the same test at 1 μg/μL, caused large areas of necrosis around the puncture point on fat hen. On other dicot (poppy, annual mercury, wild cucumber), through medium ones (tree of heaven, tomato, common sowthistle), the phytotoxicity varied from large necrotic areas to small necrotic spots (black nightshade). When compound **36** was assayed on several cultivated (wheat, oat, barley) and wild (canarygrass, slender foxtail, wild oat) monocots, it was not toxic. Both metabolites **35** and **36** did not exhibited antifungal and antibacterial activities when assayed at up to 50 μg per disk on *Geotrichum candidum*, *Pseudomonas syringae sp. syringae* and *E. coli*. and no zootoxic activity when tested, at concentrations up to 40 μg/mL in sea water on brine shrimp larvae (*Artemia salina* L.) [87,88]. The ascaulitoxin aglycone **37** was not biologically characterized, as in solution it converted into a complex mixture that was not identified, but could be probably the corresponding lactones and lactams.

The mode of action of the ascaulitoxin (**35**) was studied on *Lemna paucicostata*. Compound **35** showed a strong growth inhibition, with an IC_50_ value less than 1 μM, while assayed at 3 μM it almost completely inhibited the plant growth. Its action is slow, starting with growth inhibition, followed by darker green fronds, and then chlorosis and death. Most amino acids comprising non-toxic non-protein amino acids reversed the effect of the toxin **35** when supplied in the same medium. The addition of sucrose slightly increased the activity. D-Amino acids showed an inhibitory activity as that of ascaulitoxin, suggesting that toxic effects could not be due to the inhibition of amino acid synthesis. Oxaloacetate also reversed the activity. LC-MS analysis did not show interaction of the compound with lysine, which strongly reversed the effect of compound **35** [93].

In addition, to estimate the commercial potential of *A. caulina* toxin mixture, obtained from cation exchange chromatography of fungal culture filtrates as above reported [92], toxicity was evaluated according international protocol on aquatic and terrestrial organism as the algae *Daphnia magna*, the fish *Brachydanio rerio* and earthworms *Eisenia foetida*. The results obtained in both acute and chronic toxicity, by comparing the ecotoxicological profile of the toxin mixture with that of other herbicides, showed its lower ecotoxicity [94].

Later, chenopodolin (**38**, Figure 2), unrearranged ent-pimaradiene diterpene, was isolated from the culture filtrates of *Phoma chenopodiicola*, which is another fungal pathogen proposed as mycoherbicide for the control of *C. album*. Compound **38** caused necrotic lesions on *Mercurialis annua*, *Cirsium arvense* and *Setaria viri*de (two dicot and one monocot plant species, respectively) by leaf puncture test at a concentration of 2 mg/mL. Some of its key derivatives were prepared such as the 3-*O*-*p*-iodobenzoyl, 3-*O-*acetyl esters, 1-*O*-deacetyl-, 6,*O*,15,16-tetrahydro- and 6,*O*,7,8,15,16-hexahydro derivatives, and assayed on the same tree weeds in comparison to the parent toxin. Among the derivatives tested, only the 1-*O*-deacetyl derivative caused necrosis, but to a lesser extent than those induced by compound **38**. These results showed that the hydroxyl group at C-3, the α,β-unsaturated ketone at C-6 and probably the vinyl group at C-13 are important features for the activity [95].

Successively, three phytotoxic tetrasubstituted furopyrans such as chenopodolans A-C (**39**–**41**, Figure 2) were isolated together with (*R*)-6-hydroxymellein (**42**, Figure 2) from the same fungus [96]. When the three toxins **39**–**41** were assayed on the punctured detached leaves of *Sonchus oleraceus*, *M. annua* and *C. album*, chenopodolan B (**40**) was the most toxic compound, spreading necrosis on all three plant species tested, while chenopodolans A and C (**39** and **41**) had, respectively, almost the same toxicity, showing in particular phytotoxic activity against *S. oleraceus* and *M. annua*, and no toxicity. These results showed that the side chain linked to the pyran ring is an important structural feature for phytotoxicity. In particular, the presence of the tertiary hydroxy group present in compound **39**, which is secondary in metabolites **40**, is very important for the activity. Chenopodolan B (**40**) is the only metabolite that showed weak zootoxic activity in the assay on brine shrimps (*A. salina* L.), while any compound that showed antimicrobial activity when tested against *G. candidum* mand Gram-positive *B. subtilis* and Gram-negative *E. coli*. Chenopodolans A and B could have additive phytotoxic activities with chenopodolin and (-)-(*R*)-6-hydroxymellein produced by the same fungus [96]. 

From the same organic extract, other phytotoxic metabolites as chenopodolan D, chenisocoumarin and chenopodolin B (**43**–**45**, Figure 2) were isolated. In particular, chenopodolin B (**45**) differed from compound **38** for the acetylation of the hydroxy group at C-3, while chenopodolan D from the close metabolites **39**–**41** for the chain nature linked at C-4 of the pyran ring [97]. When assayed at 4 × 10^−3^ M by leaf puncture against non-host weeds such as *Sonchus arvensis*, *Urtica dioica* and *Parietaria officinalis*, compounds **43** and **45** showed phytotoxicity, while metabolite **44** was inactive. These results confirmed that the nature of the side chain at C-4 in chenopodolans, and in particular its hydroxylation, are important features for activity. The activity of chenopodolin B could also be explained by its possible hydrolysis to chenopodolin through the well-known lethal metabolism [53]. Successively, other new furopyrans close to compounds **39**–**41** and **43** such as chenopodolans E and F (**46** and **47**, Figure 2) were isolated from the same organic extract, and the absolute configuration of chenopodolan B, remaining unassigned, was also determined. Both compounds **46** and **47** were assayed through leaf-punctured leaves at 2 mg/mL on *S. arvensis*, and only metabolite **47** was active, though it was not toxic when applied to *Setaria*. Compounds **46** induced ca. 75% larval mortality on *A. salina* larvae at 0.1 mg/mL [98].

**Figure 3:**
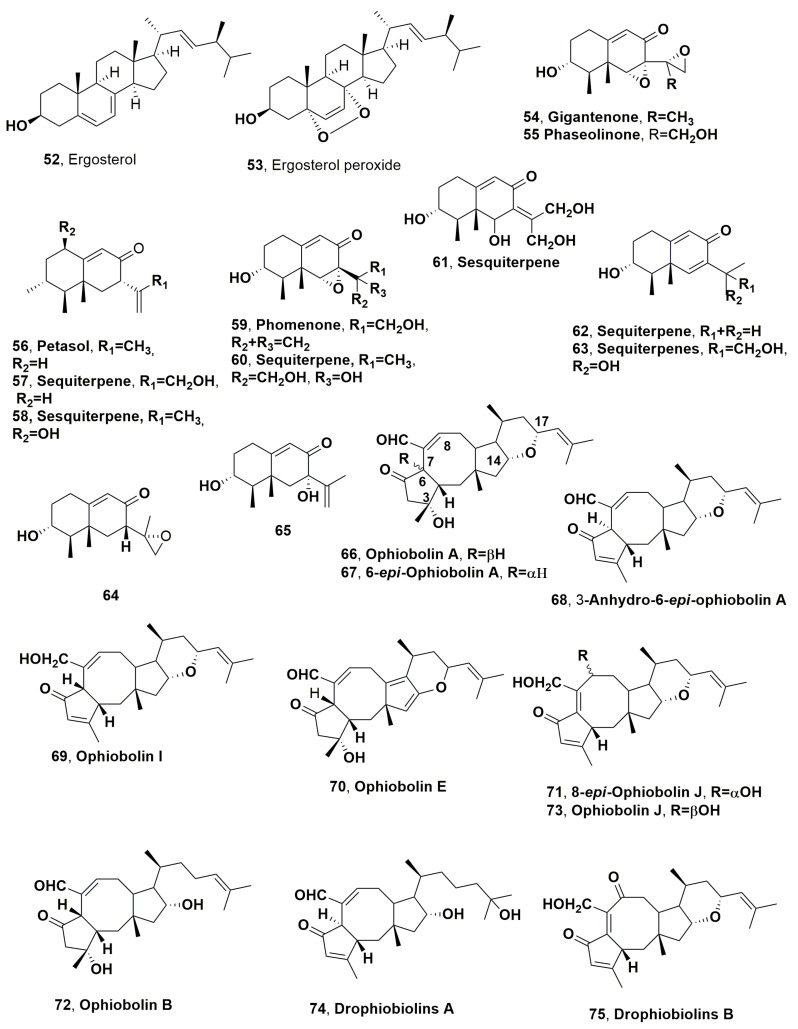
Metabolites produced by *Phomopsis convolvulus* (**52** and **53**) and *Drechslera gigantea* (**54**–**75**).

The phthalides such as convolvulanic acids A and B, convolvulol (**48**–**51**, Figure 2) and the α-pyrone convolvulopyrone (**52**, Figure 2) were isolated together with ergosterol and ergosterol peroxide (**52** and **53**, Figure 3) from the culture filtrates of *Phomopsis convolvulus*, which is a host-specific pathogen causing leaf spots and anthracnose lesions to the very dangerous and perennial weed bindweed (*Corzvolvulus arvensis*). All compounds were tested by leaf-cuttings on the host plant, and strong toxicity was observed assaying both compounds **48** and **49**, while lesser phytotoxicity was exhibited by compounds **50** and **51**. Convolvulanic acid B (**49**), at concentrations of 3.5 × 10^−4^ M, caused wilting and browning of plant tissues after only 4 h after inoculation. The same symptoms were induced by compound **48,** but at 5 × 10^−4^ M and at 12 h after inoculation. The sterols, as expected, were not toxic [99].

Twelve eremophilane sesquiterpenes (**54**–**65**, Figure 3) were isolated from the culture filtrates of *Drechslera gigantea*, a pathogen fungus of several grasses such as common weed crabgrass (*Digitaria* spp.), quackgrass (*Agropyron repens*) and Bermuda grass (*Cynodon dactylon*) [99]. Among all the sesquiterpenes isolated, there was the already known gigantenone (**54**) [100], which is close to phaseolinone (**55**), produced as the main phytotoxin by *Macrophomina phaseolina*, the causal agent of soyabean charcoal rot. This is one of the most common and severe dry root rot diseases around the world, affecting about 500 cultivated and wild plant species [101]. In addition, petasol and phomenone (**56** and **59**), which were previously obtained, respectively, from the higher plant *Petasis hybridis* [102] and from *Phoma destructive*, which is a pathogen agent of tomatos [103], were also isolated from *D. gigantea*. The other sesquiterpenes (**57** and **58** and **60**–**65**) were not associated with any common practical name. Sesquiterpenes **54**, **55**, **56** and **59,** assayed by leaf puncture on monocot species at 10–20 μmol droplet, caused green islands sized >20 mm. The latter were <10 mm or there was no effect when were tested against the other eremophilans, except compounds **58**, **60** and **61**, which induced necrosis on dicots species when tested at 10–20 μmol [104].

Ophiobolin A (**66**, Figure 3), a carbotricyclic sesterterpene, was isolated as the main phtytotoxin together with its analogues 6-*epi*-ophiobolin A, 3-anhydro-6-*epi*-ophiobolin A and ophiobolin I (**67**–**69**-Figure 3) from the culture filtrates of same fungus, using a strain collected in Florida from naturally infected large crabgrass (*Digitaria sanguinalis* L.) [105]. Ophiobolin A (**66**) shares the same 5:8:5 carbotricyclic ring system with fucicoccins and cotylenins, two other groups of microbial metabolites produced by *Phomopsis amygdali* (syn. *Fusicocum amygdali*), the causal agent of almond and peach diseases [19], and with *Cladosporium* sp. 501–7W [106]. Compound **66** is the first member of a well-known phytotoxic sesterterpene group produced by several fungi attacking cereals, such as rice, maize and sorghum, and whose structure was independently determined by Canonica et al., 1966 [107] and Nozoe et al., 1966 [108]. Later, several close sesquiterpenes were isolated including ophiobolin B from *Bipolaris oryzae* [109], ophiobolin C from *Bipolaris zizanie* [108], ophiobolin D from *Cephalosporium caerulens* [110,111] and ophiobolin F from *Bipolaris maydis* [112]. Successively, a large number of ophiobolin up to ophiobolin Z and analogues were isolated from different fungi whose mainly belonged to *Bipolaris* genus. Among these ophiobolins, there are several that showed phytotoxic activity against agrarian and weedy plants as well as antibiotic, nematocidal, antiviral and cytotoxic activities. Furthermore, several SAR studies, which were carried using ophiobolin natural analogues and hemisynthetic derivatives, several of which were prepared starting from the parent cmpound **66**, were reviewed by Au et al., 2000 [113] and Tian et al., 2017 [114]. All these results were also briefly reported in a recent review focused on the anticancer activity of ophibolin A and its mode of action [115]. Extensive studies were also carried out on ophiobolin A as a promising drug against the malignant brain glioblastoma. Among these studies, recently, a pharmacophore-directed retrosynthesis applied to ophiobolin A allowed the preparation of some bicyclic derivatives that showed anticancer activity. In particular, these synthetic derivatives exhibited cytotoxicity activity toward a breast cancer cell line (MDA-MB-231) and confirmed the importance of structural complexity for selectivity of ophiobolin A (vs. MCF10A cells), while C3 variations modulate stability [116]. Furthermore, considering the role of epithelial-mesenchymal transition (EMT) on cancer and treating cancer cells with paraptosis-inducing compounds, such as ophiobolin A (**66**), which specifically targets otherwise-insensitive CSC and EMT cells to re-sensitize bulk tumor populations to chemotherapies, it was demonstrated that EMT is a key driver in increasing sensitivity to paraptosis-induced cell death with a short-term treatment with compound **66** [117].

Ophiobolin A (**66**) and its analogues 6-*epi*-ophiobolin A and 3-anhydro-6-*epi*-ophiobolin A and ophiobolin I (**67**–**69**) were assayed on the punctured detached leaves of several monocot grasses (*Avena ludoviciana*, *Bromus sterilis* L., *Cynodon dactylon*, *D. sanguinalis* L., *Echinochloa crus-galli* L., *Oryzopsis miliacea* L., *Phoenix canariensis*, *Setaria viri*dis L.) and dicotyledon weeds (*Amaranthus retroflexus* L., *Chenopodium album* L., *Convolvulus arvensis* L., *Diplotaxis erucoides* L. and *Sonchus oleraceus* L.), and compound **66** appeared to be more phytotoxic when compared to its analogues. Thus, structural features important to phytoxicity appeared to be the hydroxy group at C-3, the stereochemistry at C-6 and the aldehyde group at C-7. Furthermore, grass weeds seemed more sensitive to the phytotoxins than dicotyledons species, which tested ophiobolin caused large necrosis even at the lowest concentration assayed [105]. Later, from the same organic extract of *D. gig*antea, both liquid and solid kernels cultures ophiobolin E and 8-*epi*-ophiobolin J (**70** and **71**, Figure 3) and ophiobolins B and J (**72** and **73**, Figure 3) were, respectively, isolated [118]. The ophiobolins **70**–**73** were tested at the concentration of 0.5 mg/mL on four weedy plants (*Avena sterilis*, *Bromus* sp., *Hordeum murinum* and *Oryzopsis miliacea*) using the leaf puncture assay. Among all compounds tested, only ophiobolins B and J (**72** and **73**) showed phytotoxicity, while compounds **70** and **71** were not toxic. In particular, ophiobolin B exhibited strong toxicity against *Bromus* sp. and *Hordeum marinum* leaves [118]. The modulated activity showed ophiobolin B (**72**) was similar to that previously observed for ophiobolin A [105], suggesting that the opening of the etheral ring between C-14 and C-17 is not important for the phytotoxicity. Furthermore, based on ophiobolin J (**73**), reduced or not toxic as showed by the close ophiobolin I (**69**) [100], this response could be attributed to the different conformation that the octacyclic B ring can assume as a consequence of the different position of the double bond, which is located between C-7 and C-8 in compound **69**, and between C-6 and C-7 in compound **73**. Furthermore, the lack of toxicity showed by compound **71** should be due to the epimerization of the hydroxy group of C-8, observed for the first time in ophiobolin group. Finally, the several structural differences present in ophiobolin E when compared to the parent compound (**66**) could justify its inactivity on all the plants tested [118].

Recent new investigation of the organic extract of *D. gigantea* culture filtrates, which were prepared to obtain more crystalline ophiobolin A (**66**) for in-depth investigation of its mode of action as an anticancer compound, another two ophibolins, named drophiobiolins A and B (**74** and **75**, Figure 3), were isolated [119]. Both ophiobolins **74** and **75**, in comparison to compound **66**, were assayed at 10^−3^ M by leaf puncture assay on cultivated (*Lycopersicon esculentum* L.), host (*D. sanguinalis* L.) and nonhost (*C. album* L.) weedy plants. Both compound **74** and **75** induced significant phytotoxicity. Drophiobolins A and B exhibited the same phytotoxicity as that of ophiobolin A on the host plant and tomato, while lesser toxicity was caused, in respect to compound **66**, on *C. album*. Decreasing the concentration at 10^−4^ M, their toxicity slightly decreases on all tested plants with compounds **74** which appeared inactive on *C. album*. Furthermore, both drophiobiolins A and B (**74** and **75**) showed cytotoxicity against Hela B cells with IC_50_ value of 10 μM, but had a lesser or nil effect against Hacat, H1299 and A431 cells when compared to that of compound **66** [119].

**Figure 4:**
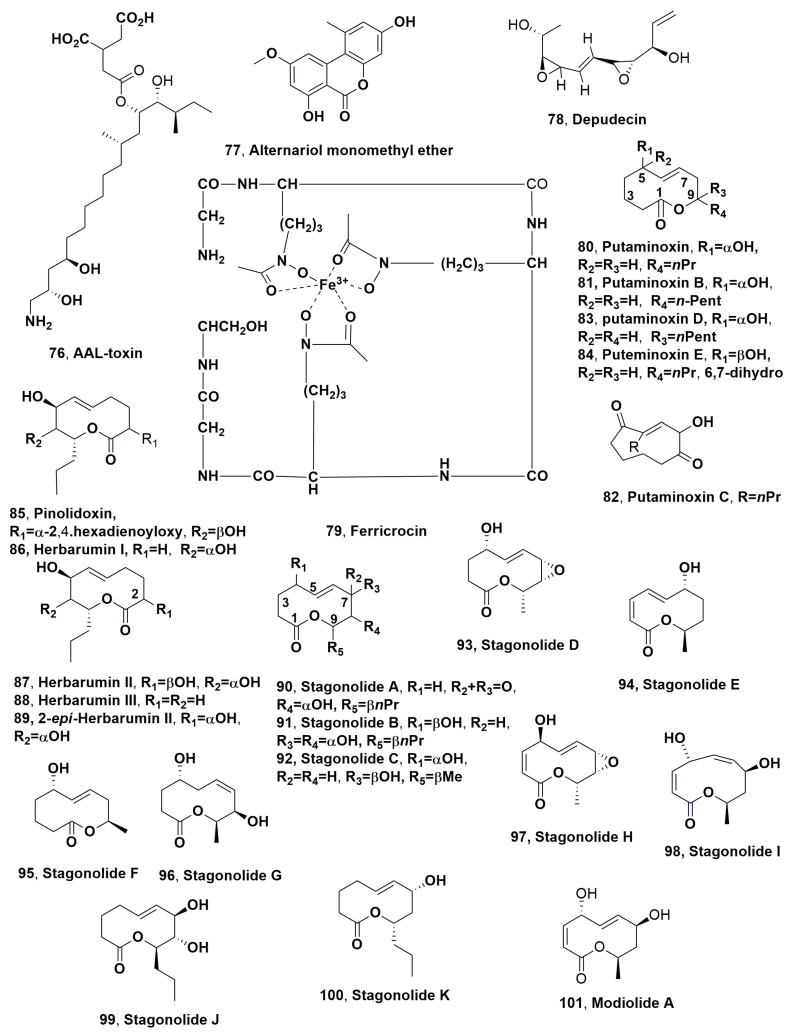
Metabolites produced by *Alternaria alternata* (**76** and **77**), *Nimbya scirpicola* and *Alternaria brassicola* (**78**), *Colletotrichum gloeosporioides* (**79**), *Phoma putaminum* (**80**–**84**), *Dydimella pinodes* (**85**), *Phoma herbarum* (**86**–**89**) and *Stagonospora cirsii* (**90**–**101**).

AAL-toxin, alternariol monomethyl ether (**76** and **77**, Figure 4) and tenuazoic acid (**1**) were isolated from a strain of *A. alternata* which was obtained from infected tomato (cv. Beefsteak) plants. Among the nine strains isolated and grown on autoclaved rice medium and corn meal agar medium, only the SWSL 1 (NRRL 18822) showed phytotoxicity on 1-week-old jimsonweed plants [120].

Different *Alternaria* species were isolated from fruits, vegetables, grains and weeds including citrus, jimsonweed (*Datura stramonium* L.), lettuce (*Lactuca saligna* L.), pear (*Pyrus communis* L.), sicklepod (*Cassia obtusifoLia* L.), anoda (*Anoda cristata* L.), sorghum (*Sorghum bicolor* L.) and wheat (*Triticum aestivum* L.). These fungi caused different diseases such as black spot of Japanese pear, tobacco brown spot, early blight of potatoes and tomatoes, chlorosis of citrus, lettuce, and tobacco, leaf spotting of sicklepod, jimsonweed and spurred anoda and stem canker of tomatoes [121,122,123,124,125,126,127,128,129,130,131,132,133]. AAL-toxin (**76**) at concentrations of 200 μg/mL induced damage on excised jimsonweed leaves, which were characterized by soft rot diffusing from the point of inoculation along the veins, adaxially or abaxially to leaves. Alternariol monomethyl ether and tenuazonic acid (**77** and **1**) at concentrations of 800 and 420 μg/mL, respectively, were inactive in the same assay [120].

Depudecin (**78**, Figure 4) was isolated from *Nimbya scirpicola*, which had been obtained from a diseased paddy field weed, *Eleocharis kuroguwai* (Japanese name: kuroguwai) [134]. The fungus producer *N. scirpicola* induced strong orange spotting on stems of kuroguwai and exhibited high host specificity. When tested by leaf puncture assay at 10 μg/dose on several weeds, including the host and agrarian plants such as kuroguwai, kidney bean, cowpea hairy beggarticks, velvetleaf, barnyardgrass, green foxtail, wheat, rice and corn, depudecin (**78**) induced serious damage on kuroguwai stems necrotic lesions (5 mm diamater), while lesser necrotic symptoms (3–4 mm in diameter) were induced on cowpea and kidney bean, but was inactive on the other plants tested. When compound **78** was in droplets applied to intact kuroguwai stems, their surface became orange in appearance and then exhibited necrosis, which are symptoms similar to those found on diseased kuroguwai in the field. Compound **78**, tested at 10^−4^, 10^−3^ and 10^−2^ M on lettuce seedling, inhibited roots at ca. 20, 81 and 100%, respectively [134]. Furthermore, depudecin was previously isolated from *Alternaria brassicicola* during a screening carried out to find new natural anticancer compounds. In fact, compound **7**8 caused the flat morphology of ras- and src-transformed NIH3T3 cells at a concentration of 1 ug/mL. Increasing its concentration (8–10 μg/mL), the 50% the growth inhibition of the same cells was observed [135].

Ferricrocin (**79**, Figure 3) is a siderophore which was isolated as phytotoxic metabolite from the culture of *Colletotrichum gloeosporioides* obtained from infected blackberry (*Rubu*s spp.) [136]. The plant, which is well known as a host of *C. gloeosporioides*, was collected in North Carolina, USA [136]. Compound **79** was also previously isolated from *Neurospora*, *Aspergillus* and *Epicoccum* cultures [137,138,139,140]. Compound **79** was assayed against cotyledons of velvetleaf (*Abutilon theophrasti*) (7 days old) inducing phytotoxicity from 10^−2^ M to 1/64 × 10^−2^ M. Furthermore, compound **79** and its derivative deferriferricrocin, which lacking any metal, was assayed by leaf-puncture assay on some weeds such as beggarweed (*Desmodium tortuosum*), dandelion (*Taraxacum vulgare*), pigweed (*Amaranthus retrofiexus*), johnsongrass (*S. halepense*), tall fescue (*Festuca arundinacea*), velvetleaf (*A. theophrasti*) and jointvetch (*Aeschynomene* spp.). The plants were treated with a 2 mL solution at 1 g/4 mL and phytotoxicity observed 7 days after the inoculation. The results obtained showed that deferriferricrocin had a stronger phytotoxic activity which appeared faster with respect to that of compound **79** [136].

Putaminoxin (**80**, Figure 4) is a phytotoxic nonenolide isolated as the main metabolite from *Phoma putaminum*, which was proposed for the biocontrol of *Erigeron annuus* [141]. This weed, which is commonly named annual fleabane, is an indigenous weed from North America widely found in field and pastures all over Europe, including Italy, on which the fungus induced necrotic spots, surrounded by chlorotic haloes. Compound **80** was tested by leaf puncture assay on the host plant at 20 μg per droplet, and induced chlorosis and 2 days later necrosis. When tested, using the same method on other weed species (annual, dog’s mercury, annual sowthistle, clover chickweed, fat-hen) and on non-host cultivated plants (mandarin, globe artichoke, nettle parsley, Swiss chard, strawberry, sweet basil and tomato), putaminoxin (**80**) showed a range of toxicities. Among the plants, tested mandarin and sweet basil appeared to be the less sensitive, while annual dog’s mercury was the most sensitive. The toxin also showed at 100 μg/disk weak toxicity toward *G. candidum* at 100 μg/disk, but was not toxic against *E. coli* and *B. subtilis* as well as against *A. salina* larvae [141]. Later, putaminoxins B and C (**81** and **82**, Figure 4), which are a phytotoxic nonenolide and a hexaketide, respectively, were isolated from the organic extract of the same fungus [142]. Both compounds **81** and **82** were tested on punctured and detached leaves of several weeds and cultivated plants at 8 μg/droplet. After 2 days, putaminoxin C (**82**) induced necrotic spots only on *M. annua* (annual dog’s mercury) and on *Cynara cardunculus* (globe artichoke) and chlorosis on *Lycopersicon esculentum*. No toxicity was observed on *Vicia faba* (faba bean), *Sorghum bicolor* (sorghum) and *Cucumis sativus* (cucumber). Putaminoxin B (**81**) did not have toxicity of any plant used. Only putaminoxin C (**82**) tested up to 40 μg/disk showed a clear inhibition of the growth of *B. megaterium* and no toxicity on *Pseudomonas* sp. [142]. Successively putaminoxins D and E (**83** and **84**, Figure 4) were isolated from the same fungus as two other nonenolides close to compound **80** [142]. When tested using the same method at concentration of 4 × 10^−3^ M on tomato and annual fleabane leaves, putaminoxin D and E (**83** and **84**) had no phytotoxic activity. These results suggested that the presence of the unaltered alkyl side chain at C-9 and both the double bond and the hydroxy group at C-5 of the macrocyclic ring are structural features important for the activity [143].

A SAR study was carried out using putaminoxin and the close pinolidoxin together with some of their natural analogs and hemisynthetic derivatives. Pinolidoxin (**85**, Figure 4) [144] is a phytotoxic nonenolide produced as the main metabolite together with it 7-*epi*, 5,6-dihydro and 5,6-epoxy analogues [144] by *Dydimella pinodes* (syn. *Ascochyta pinodes*), which is the causal agent of pea (*Pisum sativum* L.) anthracnose. The derivatives prepared from putaminoxin (**80**) were its 5-*O*-acetyl- and 5-*epi*-derivatives, while those of pinolidoxin (**85**) were its diacetyl, isopropylidene and hexahydro derivatives. The phytotoxic, antifungal and zootoxic activities of all natural nonenolides and their derivatives were assayed, respectively, on weeds such as annual fleabane, annual mercury showy crotalaria, Canada thistle, fat hen, buttercup oxalis and Noogoora burr, as well as crop plants such as sugarbeet cucumber, pea, tomato, sorghum and globe artichoke. The antifungal and zootoxic activities were assayed against *G. candidum* and brine shrimp (*A. salina* L.) larvae according to the methods previously reported [145]. Putaminoxin and pinolidoxin showed the strongest phytotoxicity, which appeared related to the integrity of the nonenolide ring and to the presence of both the hydroxy groups and the unmodified propyl side chain. All compounds exhibited antifungal activity, whereas pinolidoxin analogs and derivatives possessed high to weak zootoxicity [146].

Later, other nonenolides such as herbarumins I and II (**86** and **87**, Figure 4) were isolated from *Phoma herbarum* and proposed as mycohercides to biologically control the very dangerous weed prince’s feather (*Amaranthus hypochondriacus*). Compound **86** and **87** inhibited seed germination and seedling growth of *A. hypochondriacus*, showing IC_50_ values of 5.43 × 10^−5^ and 1.25 × 10^−4,^ respectively. Thus, herbarumin I (**86**) appeared to be more active than compound **87** and the positive control (2,4-D: 2,4-dichlorophenoxyacetic acid) [147]. Later, herbarumin III (**88**, Figure 4) was isolated from the organic extract of the same culture filtrates, which exhibited strong phytotoxicity against seedlings of *A. hypochondriacus* with IC_50_ value of 2 × 10^−5^ M, inhibiting the radicle growth stronger than 2,4-D used as positive control and compound **87**, while its activity was similar to that of herbarum I (**86**). These results suggested that the presence of the hydroxy group at C-2 decreases the phytotoxic activity [148]. The 2-epimer of herbarumin II (**89**, Figure 4) was isolated together with (*E*)-5,9-dihydroxydodec-6-enoic acid and herbarumin II (**87**) from the culture filtrates of *Paraphaeosphaeria recurvifoliae*, which were obtained from the leaf lesions of pendulous yucca (*Yucca recurvifolici*) in Korea [149]. Herbarumin I and III (**86** and **89**) and (*E*)-5,9-dihydroxydodec-6-enoic acid, when assayed at a concentration of 1.3 mg/mL on murine tyrosinase, partially purified, showing weak inhibitory activity such as 30%, 17% and 12%, respectively, while the positive control kojic acid exhibited 85% of inhibition tested at the same concentration [149]. More recently, herbarumin II ant its 2-epimer (**87** and **89**) were isolated together with pinolide, another close nonenolide, and pinolodoxin (**85**), which was the main metabolite, from a more virulent strain of *D. pinodes* [150]. When all these latter nonenolides were tested on several weeds and crops plants, only pinolidoxin showed phytotoxicity, suggesting an important role played from the hydroxy group at C-7 as well as its configuration. In fact, in pinolide, which had null or weak activity, this hydroxy group is α-located, while in pinolidoxin, herbarumin I and II and 2-*epi*-herbarumin II (**85**, **86**, **87** and **89**), which showed a strong activity, the same hydroxy group had β-configuration. The configuration of the hydroxy group at C-2 seems to assume a minor importance. In fact, in all the above nonenolides, it is α-located except for herbarumin II, in which this hydroxy group is β-located, and this different configuration did not affect the phytotoxicity. Finally, the 2,4-hexadienoic acid esterification of the hydroxy group at C-2, present only in pinolidoxin (**85**) seems a very important feature for its phytotoxicty [150]. 

Other nonenolides, named stagonolide A [151] and B–F [152], G–I [153] and J and K [154] (**90**–**100**, Figure 4) and modiolide A [153] (**101**, Figure 4), were isolated from *Stagonospra cirsii*, which was proposed as a mycoherbicide for the control of Canada thistle (*Cirsium arvense* L.). This is a perennial and very dangerous weed that grows on arable land in North America, New Zealand and Europe, including the European part of the Russian Federation [155,156]. Stagonolide (**90**) was assayed on the leaves of different plant species such as lettuce, zinnia, sow-thistle, sunflower, pepper, peppermint, radish, hollyhock, pea and wheat, and annual flaebane induced high sensitivity the host plant Canada thistle as well as several of the other weeds, while two species of Solanaceae were insensitive to it. Compound **90** was tested on *C. arvense* seedling roots since 1 × 10^−4^ M caused phytotoxic symptoms that increased at a concentration of 5 × 10^−3^ M, becoming necrosis when it reached ~4 mm in diameter 48 h after treatment. When tested at the same concentration on leaf discs of weed species and cultivated plants, stagonolide (**90**) did not showed specificity. At concentrations ≥1 × 10^−6^ M, the nonenolide (**90**) inhibited the growth of *C. arense* seedling roots and decreased their length more than 30%. While tested at 1 µg/mL (~5 × 10^−6^ M) on seedlings of different plant species, it was more effective regarding the Asteraceae species (about 70% inhibition) than those of wheat and radish seedlings (about 30% inhibition). Finally, cucumber seedlings were insensitive to stagonolide at this concentration. The toxin showed low toxicity and no toxicity against *Colpoda steinii* (Protozoa) when tested at concentrations of 2 × 10^−4^ M and 1 × 10^−4^ M, respectively. At the concentration of 50–100 µg of stagonolide/disc, compound **90** showed weak antimicrobial activity against the fungus *Candida tropicalis*, but was not toxicit on *B. subtilis* and *E. coli* [147]. When stagonolides B-F (**91**–**95**) were assayed at a concentration of 1 mg/mL by leaf disk puncture assay against *C. arvense* and *Sonchus arvensis*, they appeared to not be toxic [152]. Nonenolides **96**–**101** were tested at 1 mg/mL, and metabolite **97** appeared to be the most toxic against *C. arvense* leaves. Stagonolide I and modiolide A (**98** and **101**) were markedly less active, while stagonolide G (**96**) was no toxic. When tested at the same concentration on cultivated plants, only stagonolide H (**97**) inhibited root growth in chicory seedlings (85% compared to control), and induced different sensitivity to the leaves of eight plant species, while other compounds were inactive at the concentration used [153].

When stagonolides J and K (**99** and **10**0) were assayed at 0.13–2.00 mg/mL by punctured leaf disks of the host plant (*S. arvensis*), *C. arvense* and *E. repens*, compound **99** was non-toxic, while significant necrotic lesions (about 2 mm in diameter) were observed for compounds **100** at the maximal concentration of 2 mg/mL (∼10 mM) 2 days after the treatment, though it did not showed phytotoxicity against *E. repens* leaves [154]. 

Successively, stagonolides A, J and K, herbarumin I (**90**, **99, 100** and **86**), and some their hemisynthetic derivatives, which were prepared through the parent compounds chemical modifications, such as 8-acetylherbarumin I, bis(acetyl)herbarumin I, 7-acetylstagonolide J, bis(acetyl)stagonolide J, acetylstagonolide K, 8-*O*-acetylstagonolide A and C-7 oxidized stagonolide K, were used to carry out a SAR study focused on recognizing structural features important to the design of new herbicide. The phytotoxicity of all natural stagonolides and their derivatives were assayed by leaf puncture assay on *S. arvensis*, agar seedling assay using lettuce (*Lactuca sativa*), and microalgae assay using *Haematococcus lacustris* as well as the antimicrobial activity using *B. subtilis*, and the cytotoxic activity using Sf9 cell line (ECACC 89070101) of the fall armyworm (*Spodoptera frugiperda*). The results obtained showed that stagonolide A (**90**) and C-7 oxidized stagonolide K showed the strongest phytotoxic activity in leaf puncture assay and agar seedlings assay, and that the oxidation of C-7 hydroxyl group as in **90** and acetylstagonolide A determined toxicity regarding microalgae, *B. subtilis* and Sf9 cells independently from the configuration of C-9 propyl chain, which is *R* in **99** and its 8-*O*-acetyl derivative and *S* in oxidized C-7 stagonolide K. The compounds which did not showed oxidized C-7 exhibited none or little non-target activity, while the compounds having a 7*S* configuration possessed higher phytotoxicity than their 7*R* analogues. The mono- and bis(acetyl)-derivatives of herbarium I (**86**) showed high inhibitory activity against seedling growth and the lack of side toxicity appeared to have a strong potential for the development of pre-emergent herbicides [157].

**Figure 5:**
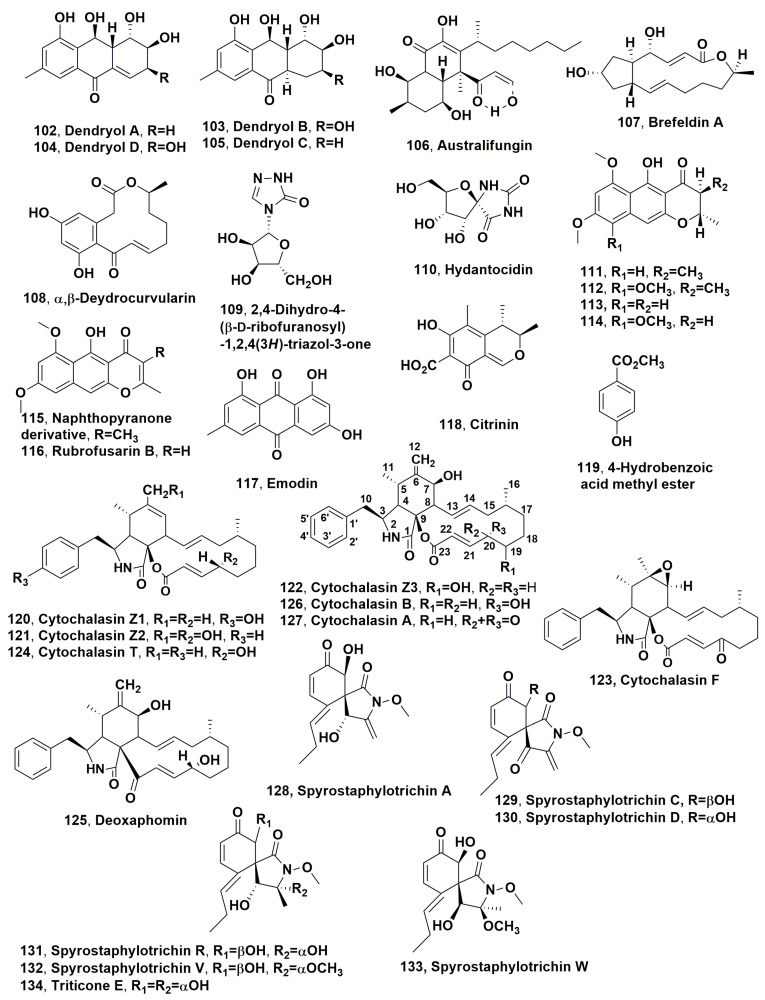
Metabolites produced *Dendryphiella* sp. (**102**–**105**), *Sporormiella australis* (**106**), *Alternaria zinniae* (**107** and **108**), *Actinomadura* sp. (**109**), *Streptomyces hygroscopicus* (**110**), *Guanomyces polythrix* (**111**–**119**), *Pyrenophora semeniperda* (**120**–**126** and **127**–**134**).

Dendryols A-D (**102**–**10**5, Figure 5), which are anthraquinones derivatives, were isolated from the weed pathogen *Dendryphiella* sp., which was obtained from diseased *Eleocharis kuroguwai* [158]. The phytotoxic activity of compounds **102**–**105** was tested by leaf puncture assay against 10–14-day-old host plant kuroguwai and other weeds and crop plants such as barnyardgrass, velvetleaf, rice corn and cowpea at concentrations starting from 5 mg/μL [159]. 

After 5 days of incubation, all compounds induced phytotoxic symptoms on the host plant kuroguwai. Among all the plant tested, barnyardgrass appeared to be sensitive to the four compounds showing necrotic area spread from the applied point to the top of the leaf [159].

Australifungin (**106**, Figure 5) was isolated from *Sporormiella australis* and belonged to a new group of ceramide synthase inhibitor. Thus, compound **106** was used as a tool to study ceramide synthase inhibition as a phytotoxic mechanism comparing its toxicity with that of sphingosine analogues using model systems for phytotoxicity and mammalian toxicity. Australifungin (**106**) inhibited the ceramide synthetase enzyme as the unrelated mycotoxins-AAL-toxins and fumonisin B in the sphingolipid biosynthesis pathway of animals. When tested at 5 μM on duckweed (*Lemna pausicostata*), cultures of australifungin (**106**) induced the accumulation of the phytosphingosine and sphinganine, which are sphingolipid precursors, but less than AAL-toxin TA or fumonisin B1 used at 1 μM. These effects determined the increase in electrolyte leakage at 24 h with consequent aggregation of duckweed fronds [160].

Brefeldin A and α,β-dehydrocurvularin (**107** and **108**, Figure 5) were isolated from *Alternaria zinniae*, proposed for the biocontrol of *Xanthium occidentale* [161]. This is a widespread noxious weed which caused severe losses to Australian summer crops and pastures. Both compounds **107** and **108** were also produced as two bioactive metabolites by other fungi belonging to the genera *Alternaria*, *Ascochyta*, *Penicillium*, *Curvularia*, *Cercospora* and *Phyllosticta*. When brefeldin A (**107**) was applied at 0.3 mg/droplet to *X., occidentale* caused the appearance of wider necrosis 1–2 days after inoculation both on host leaves and cotyledons. In addition, it did not show phytotoxicity on non-host plants including weedy and cultivated species. In the leaf puncture assay, both compounds **107** and **108**, tested at 10^−4^ and 10^−3^ M, respectively, induced necrotic spots. Tijdeen et al., 1983 [162] had previously suggested that brefeldin A could be involved in the suppression of the defence mechanism of *Carthamus tinctorius* against the pathogen *Alternaria chartami*, as compound **107**, at very low concentrations, suppressed the accumulation of phytoalexins in safflower suspension cultures. Thus, a possible practical application of brefeldin A could be its use together with the fungal producer to overcome the defence reaction of *X. occidentale* and consequently increase the phytotoxic effect and the fungal efficacy to control this weed [161]. 

A new ribofuranosyl triazolone, 2,4-Dihydro-4-(β-D-ribofuranosyl)-1,2,4(3*H*)-triazol-3-one (**109**, Figure 5), was isolated as the main phytotoxin produced by a fungal strain closely resembled *Actinomadura madurae*. Although metabolite **109** is reported for the first time as a naturally occurring compound, as some its analogues [163] it was already known as synthesized compound [164]. Compounds **109** share the same target site with hydantocidin (**110**, Figure 5), which is a well-known herbicides. The last one was first isolated from *Streptomyces hygroscopicus* [165] and then successively from a number of different *Streptomyces* strains [166,167]. Then, the synthesis of compound **110** was realized also from different companies [168,169,170,171]. Triazolones were tested using foliar-applied (postemergence) and soil-applied (preemergence) applications on whole plants and seeds of several species such as sunflower (*H. annuus*), morningglory (*Ipomoea hederacea*), velvetleaf (*Abutilon theophrasti*), pigweed (*Amaranthus retroflexus*), barnyardgrass (*Echinochloa crusgalli*), giant foxtail (*Setaria faberi*), wild oats (*Avena fatua*) and blackgrass (*Alopecurus myosuroides*). Compound **109** exhibited a broad spectrum of phytotoxicity in the greenhouse, inducing phytotoxic symptoms such as stunting, systemic chlorosis and necrosis of apical meristems on all assayed species. In the postemergence test at 500 g ha^−1^, it also showed strong activity on all species except on *Amaranthus retroflexus*. In preemergence applications, the phytotoxicity was less extended. Furthermore, the rybofuranosyl triazolone **109** tested at 0.2 μM inhibited the root growth of *Arabidopsis thaliana*, but its effect was strongly reduced when tested together with adenine and guanine reaching IC_50_ value of 11.5 µM. These results suggested that adenine reversed the effects of compound **109**, while guanine had no effect. A similar effect of adenine was reported for the phytotoxicity of the close hydantocidin (**110**) [168].

Five naphthopyranone derivatives (**111**–**115**, Figure 5) were isolated, together with the well-known rubrofusarin B, emodin, citrinin and 4-hydroxybenzoic acid methyl ester (**116**–**119**, Figure 5) from the coprophilous fungus *Guanomyces polythrix* [172]. The fungus was isolated from bat guano in México in 1979. All the compounds isolated (**111**–**119**) caused significant inhibition of radicle growth of two weed seedlings (*A. hypochondriacus* and *Echinochloa crusgalli*), with IC_50_ values ranging from 1.3 × 10^−5^–1.8 × 10^−4^ and 4.0 × 10^−5^–8.8 × 10^−4^, respectively, and interacting with both spinach and bovine brain calmodulins [172]. Citrinin (**118**) was previously isolated from *Stagonospora apocyni*, which caused a leaf spot disease on hemp dogbane (*Apocynum cannabinum* L.). The fungus also produced other very well-known phytotoxin such as mellein, tyrosol and α-acetylorcinol. When placed on leaves of hemp dogbane and eight other weed species such as *Sida spinosa* L .(prickly sida), *C. album* L. (Lamb’s quarters), *Ipomoea* sp. (morning glory), *Datura stramonium* L. (jimson weed), *Sorghum bicolor* L. (sorghum); *S. halepense* L. (Johnson grass), *Nasturtium officinale* (water cress) and *Cassia obtusifolia* L. (sickle pod), all the compounds showed non-specific phytotoxicity [173].

Cytochalasins Z1, Z2 and Z3 (**120**–**122**, Figure 5) were isolated together with cytochalasins F, T, deoxaphomin and cytochalasins B (**123**–**126**, Fiure 5) from the wheat culture of *Pyrenophora semeniperda*, proposed as mycoherbicide to control grass weeds [174]. The fungus infects seeds and leaves of over 35 genera of grasses [175]. On brome grass (*Bromus* spp.) and wheat (*Triticum aestivum* L.), *P. semeniperda* caused the death of seed primordia and subsequent abortion of seed [176]. Cytochalasins are a large group of fungal natural compounds well known for their toxic effect, primarily on the mammalain cells. Cytochalasin B (**126**), which commonly occurred in other fungi, is the main metabolite produced by *P. semeniperda*, and together with the close cytochalasin A (**127**, Figure 5), are the first two members isolated of this family [177]. Cytochalasin A was also produced together with other ones above cited and non-toxic phomachalasins A-D. From *Phoma exigua* var*. exigua*, another fungus was proposed for the biocontrol of *C. arvense* and *S. arvensis* [178,179]. Today, more that 60 cytochalasins were characterized for their chemical and biological properties and subgrouped according to the size of the macrocyclic ring and residue attached to the C-3 of perhydroisoindolyl-1-one residue [26]. All the compounds isolated (**120**–**126**) were tested in seedling assays on wheat and tomato, and the most active compounds were cytochalasin B (**126**), its 21,22-dihydroderivative, cytochalasins F (**123**) and Z3 (**122**) and deoxaphomin (**125**). The same compounds reduced the root length by about 50%, and in the leaf-puncture assay, only deoxaphomin induced small necrotic lesions. These results are in agreement with those of the previously described SAR studies in which the important role of the hydroxy group at C-7 for the biological activty was already highlighted [180,181,182].

**Figure 6:**
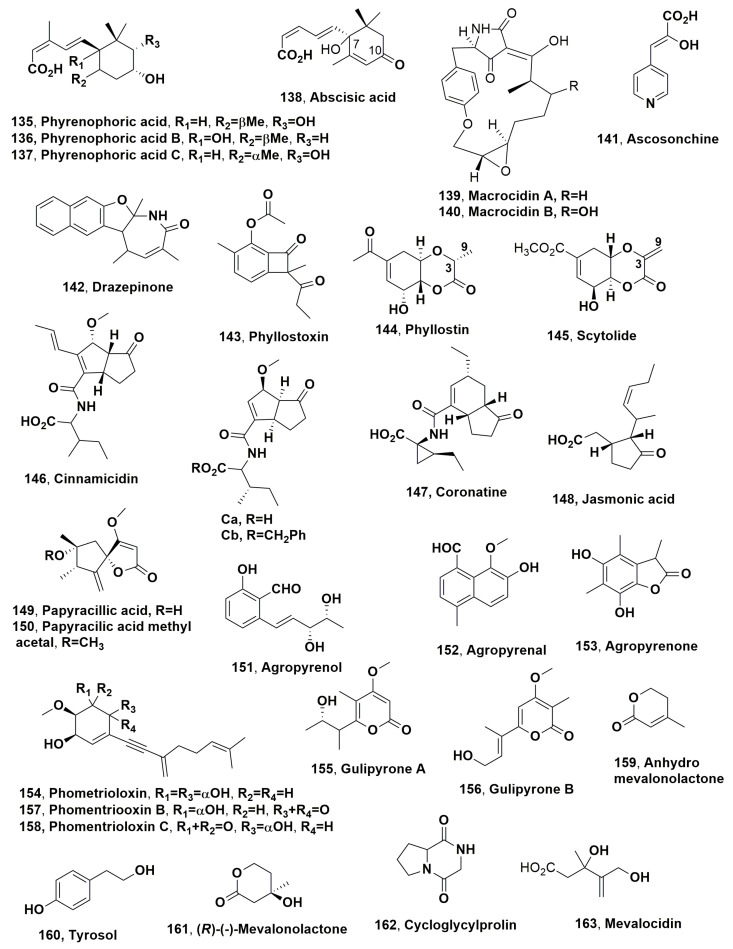
Metabolites produced *Pyrenophora semeniperda* (**135**–**138**), *Phoma macrostoma* (**139** and **140**), *Ascochyta sonchi* (**141**) *Drechslera siccans* (**142**), *Phyllosticta cirsii* (**143** and **144**), *Scytalidium uredinicola* (**145**), *Nectria* sp. (**146**), *Pseudomonas syringae* (**147**), *Ascochyta agropyrina* var. nana (**149–153**), *Phomopsis* sp. (**154**), *Diaporthe gulyae* (**155–158**), *Alternaria euphorbiicola* (**159–162**) and *Coniolariella* sp. (**163**).

The same cytochalsins were produced from strains of *P. semeniperda* isolated from cheatgrass (*Bromus tectorum*) in Utah, USA, with cytochalasin B proving to be the main phytotoxic metabolites. Cheatgrass, also known as downy brome, is an exotic winter annual grass which causes heavy losses in intensive agriculture, particularly in winter cereal production [183]. This very dangerous weed has also invaded millions of hectares of semiarid rangeland in western North America, and also induces wildfires with consequent heavy economic and environmental costs [184]. *P. semeniperda* is also able to produce phytotoxins belonging to another class of natural compounds. In fact, six spirocyclic γ-lactam, namely spirostaphylotrichin A, C, D, R, V and W (**128**–**133**, Figure 5) and triticone E (**134**, Figure 5), were produced by the same strain of *P. semeniperda*, but grown in liquid cultures [185]. Two different bioassays were carried out to test all the metabolites isolated, but compounds **129** and **130**, as well as **131** and **134**, were assayed as a mixture. Spyrostaphylotrichin A (**128**) tested at 10^−3^ M strongly reduced the cheatgrass coleoptile elongation to 33%, the mixture of compound **129/130** showed intermediate activity, while the other mixture **131**/**134** was non-toxic, and spirostaphylotrichins **132** and **133** exhibited mild toxicity. Furthermore, spirostaphylotrichins A, C and D (**128**–**130**), using leaf puncture assay, induced the appearance of necrosis, while the other compounds were inactive [185]. Successively from the same fungus but grown in solid culture were isolated pyrenophoric acid and pyrenophoric acids B and C (**135**–**137**, Figure 6), and the closely related abscisic acid (**138**, Figure 6) [186,187], which is a well-known plant hormone. The four metabolites **135**–**138** tested by *B. tectorum* seedling bioassays at 10^−3^ and 10^−4^ M caused 5 days coleoptile and radicle reductions. Abscisic acid (**138**) exhibited the strongest phytotoxicity inhibiting the germination, delaying the germination of germinated seeds, completely suppressing coleoptile elongation and markedly reducing radicle length. The observed relative toxicity ranking of the four compounds was the following: abscisic acid ≫ pyrenophoric acid B > pyrenophoric acid > pyrenophoric acid C (**138** ≫ **136** > **135** > **137**). These results suggested that the presence in C-10 of the α,β-unsaturated ketone in compound **138** seems to have an important role in the germination-inhibiting activity. The absence of this moiety in the other three metabolites significantly reduced their toxicity. Additionally, the different α-configuration of the chain bonded at C-7 observed in abscisic acid (**138**) could play a role in its strong phytotoxicity, as in the other three the configuration of the same carbon is opposite [186,187]. Among the pyrenophoric acids, as compound **136** was the most phytotoxic, its mode of action was studied. The results obtained showed that compound **136** activates the abscisic acid (ABA) signaling pathway in order to inhibit seedling establishment, and it was hypothesized that *P. semeniperda* affected plant ABA biosynthesis as a strategy to reduce seed germination, increasing its capacity to induce seed mortality and thereby increasing its virulence through higher reproductive success [188].

Macrocidins A and B (**139** and **140**, Figure 6) were produced by *Phoma macrostoma* isolated from diseased Canada thistle (*C. arvense*) growing in several geographically diverse regions [189]. The symptoms of the disease were leaves bleaching and chlorosis. Biological assay of the two compounds **139** and **140** were carried out on greenhouse-grown plants, applying each compound in post-emergence to one pot containing four species such as sunflower (*H.annuus*), giant foxtail (*Setaria faberi*), ivy leaf morning glory (*Ipomoea hederaceae*), wild oat (*Avena fatua*) or barnyard grass (*Echinochloa crusgalli*).

Both macrocidins after 10 days induced significant toxicity on the broadleaf weeds but not activity on the grass weeds [189].

Ascosonchine (**141**, Figure 6), which is a phytotoxic enol tautomer of 4-pyridylpyruvic acid, was isolated from the culture filtrate of *Ascochyta sonchi*. The latter microorganism is a leaf pathogen proposed as another potential biocontrol agent of *S. arvensi*s. Compound **141**, already assayed at 1.2 × 10^−3^ M by the leaf puncture assay on the host plant after 2 days, caused necrosis similar to those induced by the pathogen. Then, it was tested at 15 μg/droplet. Using the same methods on several weedy and cultivated plants showed interesting selective toxicity. In fact, compound **141** was completely non-toxic on all the solanaceous species (tomato, eggplant, red pepper, potato), slightly active or almost inactive on leguminous (bean and chickpea) and cucurbitaceous (melon and zucchino) plants, but induced significant necrosis on many other species, such as *Euphorbia*, *Salvia*, *Valerianella* or *Triticum* [190].

Drazepinone (**142**, Figure 6), which is a trisubstituted naphthofuroazepinone, was isolated from *Drechslera sicca*ns, which is a pathogen fungus obtained from diseased seeds of ryegrass (*Lolium perenne*), another dangerous grass weed. Compound **142** was applied at 2 μg/μL to the wounded leaves of different weeds and durum wheat, causing necrosis on almost all the species tested. The severity of necrosis ranged from very wide, as in those induced on *Urtica dioica*, to smaller ones, as those observed on *S. viridis* and *L. perenne* leaves. Also significant were the necrosis caused on *Euphorbia helioscopia*, *M. annua* and *C. album* leaves. Compound **142** did not induce toxicity on *Amaranthus retroflexus* and *Bromus* sp. [191].

Phyllostoxin and phyllostin (**143** and **144**, Figure 6), which are a pentasubstituted bicyclo-octatrienyl acetic acid ester and a pentasubstituted hexahydrobenzodioxine carboxylic acid methyl ester, respectively, were produced by *Phyllosticta cirsii*, a fungal pathogen isolated from diseased *C. arvense* leaves and proposed for the biocontrol of this dangerous weed [192]. Compound **143**, tested by leaf puncture on *C. arvense* at 10^−3^ M (20 µL/droplet), caused large necrosis, while on the contrary, compound **144**, assayed at the same concentration, was not toxic. These results are not surprising, considering the noteworthy structural differences between the two compounds, suggesting that the presence of active functional groups in phyllostoxin are not present in the other metabolites. Both compounds did not show fungicide, bacteriocide and zootoxic activity [192]. The C-3-C-9 didehydro derivative of compound **144** was also successively isolated from the same organic extract and identified as scytolide (**145**, Figure 6), previously isolated from *Scytalidium uredinicola*, which is a destructive hyperparasite of western gall rust, and *Endocronartium harknessii*, one of the most severe forest tree diseases in Canada. Compound **145** strongly inhibited the germinaion of *E. harknessii* spores [193].

Cinnacidin (**146**, Figure 6), which is a cyclopentalenone-isoleucine derivative, was isolated from *Nectria* sp. [194]. Considering the phytotoxicity of compound **146** and its potential as a bioherbicide along with its instability and low amount available, two derivatives (**Ca** and **Cb**, Figure 6) were prepared by total synthesis, both as a mixture of two diastereomers, and used in the bioassay together with the parent compound, its biosynthetic precursor coronatine (**147**) and the related jasminic acid (**148**). Coronatine and cinnacidin (**146)** carbon skeletons are very similar, but they are produced by widely different organisms, as coronatine is produced by the bacterium *Pseudomonas syringae*, while cinnacidin is produced by the fungus *Nectria* sp. All the compounds were applied on in pre- and post-emergence to the following weeds: *Avena fatua* L. (wild oat), *A. retroflexus* L. (redroot pigweed), *E. crus-galli* L. (barnyardgrass), *Ipomoea hederacea* L. (morningglory), *Setaria faberi* (giant foxtail) *Helianthus annuus* L. (common sunflower), *Alopecurus myosuroides* (blackgrass), *Abutilon theophrasti* L. (velvetleaf) *D. sanguinalis* L. (large crabgrass), *Xanthium strumarium* L. (common cocklebur), *Zea mays* L. (corn), *Brassica napus* L. (oilseed rape), *Oryza sativa* L. (rice), *Triticum aestivum* L. (wheat), *Sorghum bicolor* L. (sorghum). The phytotoxicity of the first analogue **Ca** had a result identical to that of its benzyl-protected ester (analogue **Cb**). Thus, considering the higher foliar absorption of **Cb**, due to its increased lipophilicity, the ester was used for the biological characterization and proved to be a potent herbicide. Taking into account the disease symptoms displayed by treated grass and dicot species, the mode of action of analogue of the **Cb** appears very similar to that of coronatine and jasminic acid (**147** and **148**, Figure 6), which is a very well-known bacterial toxin produced by different species of *Pseudomonas syringae* [195] and a well-known plant hormone [196], respectively. The phytotoxic activity of the ester **Cb** differed from that coronatine for the effect induced on the different plant species. Coronatine showed greater activity against warm season grasses, while **Cb** was more efficacious against cool season grasses [194]. 

Papyracillic acid (**149**, Figure 6) was isolated as the main phytotoxin produced by *Ascochyta agropyrina* var. *nana* proposed for the biocontrol of quack grass (*Elytrigia repens*), which is a noxious perennial weed widespread through the cold regions of both hemispheres [197]. When isolated as a crystalline compound, papyracyllic acid (**149**) is a stable compound that in solution converted into different isomers. Previously, it was isolated together with its methyl acetal (**150**, Figure 6), which was named papyracillic acid B, palmarumycins and microsphaeropsins from *Microsphaeropsis* sp. This latter fungus was obtained from a branch of the tree *Larix decidus* [198]. Compound **149** was also previously isolated together with lachnumon and mycorrhizin A from *Lachum papyraceum*, which produced nematicidal and antimicrobial metabolites [199]. Paypracyllic acid was also converted in some semisynthetic derivatives to perform a SAR study aimed to find a derivative with increased phytotoxicity and specificity. Compound **148** was converted in its methyl ester, methyl acetal (**150**) and four monoacetyl derivatives, two of which acted as a mixture of two inseparable epimers, and the dihydro derivative. The parent compound (**149**), its natural analogues (**150**) and the semisynthetic derivatives were tested by leaf disk puncture assay, at the concentration of 1 mg/mL, on the host and nonhost plants such as Canada thistle (*C. arvense*), Asian dock (*Rumex confertus*), dandelion (*Taraxacum officinalis*), barley (*Hordeum vulgare*), timothy grass (*Phleum pretense*), fat hen (*C. album*), double cinnamon rose (*Rosa cinnamomea*), perennial sowthistle (*S. arvensis*), hemp (*Cannabis sativa*) and red clover (*Trifolium pretense*). Compounds **149** and **150** and all the four monoacetyl derivatives showed phytotoxicity against *E. repens*, but the other derivatives were lesser toxic than papyracillic acid. Canada thistle leaves showed to be more sensitive to the same derivatives than compound **149.** These results suggested that the butanolide ring is an important feature to impart phytotoxic activity. The methyl ester of **149** was not toxic being the only one having the opening of the hemiacetalized 1,6-dioxospiran. The lack of phytotoxicity of the dihydroderivative of **149**, in which the butanolide ring is unaltered, also showed that the exocyclic methylene group at C-5 play a role in the induction of toxicity [197]. Agropyrenol, agropyrenal and agropyrenone (**151**–**153**, Figure 6) were successively isolated from the organic extract of the same fungus (*A. agropyrina* var. *nana*), and are a phytototoxic substituted salicylaldehyde, a substituted naphthalene carbaldehyde and a pentasubstituted 3*H*-benzofuranone, respectively [200]. When assayed on the leaves of some weeds such as *M. annua*, *C. album* and *S. viridis*, agropyrenol showed strong phytotoxicity, causing the appearance of necrotic lesions, agropyrenal was less active, while agropyrenone was not toxic. None of the compounds showed antibiotic, fungicidal or zootoxic activity [200]. Six derivatives of agropyrenol were prepared by chemical modification of its functional group, namely by diacetylation, chetalization and mono-oxidization of the glycol system of the side chain, the hydrogenation of the double bond of the same residue and successive diacetylation of the glycol, and by reduction to primary alcohol of the aldehyde group. Agropyrenol and its six derivatives were assayed at 2 mg/ mL by applying 20 μL of solution to detached against *C. album*, *C. arvense*, *M. annua*, and *S. ole*raceus and one monocot *S. viridis*. Agropyrenol and its derivatives were also tested to inhibit seed germination of *S. viridis* and the rootlet growth using tomato seeds. Briefly, the results obtained showed that both the double bond and the diol system of the 3,4-dihydroxypentenyl side chain as well as the aldehyde group are important features to induce phytotoxicity [201].

Phomentrioloxin (**154**, Figure 6), a phytotoxic geranylcyclohexenetriol, was isolated from the liquid culture of *Phomopsis* sp., which is a fungal proposed for the biological control of *Carthamus lanatus*. This is a widespread and troublesome thistle (*C. arvense*) which causes severe crop and pastures losses in Australia. When tested by leaf puncture at a concentration of 6.85 mM on *C. lanatus*, *C. album*, *C. arvense*, *M. annua*, *S. olerace*us and *S. viridis*, compound **154** causes the appearance of necrotic spots. Compound **154** also induced the reduction in growth and chlorophyll content in *Lemna minor* fronds and the inhibition of tomato rootlet elongation [202]. Succesively, phomentioloxin (**154**) was isolated together with gulypyrones A and B (**155** and **156**, Figure 6), two phytotoxic trisubstituted α-pyrones and phomentrioloxins B and C (**157** and **158**, Figure 6) from the cultures filtrates of a virulent strain of *Diaporthe gulyae* [203]. Metabolites **157** and **158** are, respectively, the 1,*O*- and 2,*O*-dehydro derivatives of phomentrioloxin. The fungus was obtained from stem cankers of sunflower and known to be pathogenic to saffron thistle. Other well-known metabolites were also isolated including 3-nitropropionic, the main metabolite, succinic, and *p*-hydroxy- and *p*-methylbenzoic acids, *p*-hydroxybenzaldehyde and nectriapyrone. Among all the isolated compounds, which were assayed at 5 mM on punctured leaf disks of weeds such as *Papaver rhoes*, *Ecballium elaterium*, *Urtica dioica* and *Hedysarum coronarium*, only nitropropnic acid induced significant necrosis and smaller ones also on *M. annua*, *Lactuca serriola*, *Ailanthus altissima* and *Inula viscosa*. Phomentrioloxin B (**157**) caused small but clear necrotic spots on a number of plant species, whereas gulypyrone induced leaf necrosis on *H. annuus* plantlets. All other compounds were weakly active or inactive [203].

Anhydromevalonolactone, tyrosol, (R)-(–)-mevalonolactone and cycloglycylproline (**159**–**162**, Figure 6) were isolated from of the fungus *Alternaria euphorbiicola*, a pathogen of wild poinsettia (*Euphorbia heterophylla*) [204]. This species is a major weed in many tropical and subtropical countries, and determines significantly agricultural losses in important crops, particularly soybeans and corn [205]. All the compounds isolated (**159**–**162**) were tested at concentrations as low as 80 µM by a leaf-punctured host plant causing bleached lesions with dark brown margins. When tested at 1 mM using the same method on different weeds such as *Bidens pilosa*, *Bidens subalternans*, *Brachiaria decumbens*, *Chamaesyce hirt*a and *Ipomoea grandifolia* on the leaves of other relevant weeds, (*R*)-(–)-mevalonolactone (**161**) was active only against *I. grandifolia*, with symptoms similar to those induced on the host plant. Anhydromevalonolactone and tyrosol (**159** and **160**) showed a wider spectrum of phytotoxicity. Finally, cycloglycylproline (**162**) showed no toxicity against other species, thus presenting selective activity against *E. heterophylla* [205]. As cycloglycylproline (**162**) belongs to the same group of cyclodipetides reported above as phytotoxic metabolites isolated from *A. alternata*, the causal of black leaf blight of spotted knapweed, its phytotoxic activity was not surprising.

Mevalocidin (**163**, Figure 6) is a phytotoxin isolated *Coniolariella* sp. It was also obtained by basic treatment for 2 h at 25 °C starting from methylidenemevalonlactone [206]. Compound **163** showed a broad spectrum of post-emergent herbicidal properties greater than 50 % injury than all of the broadleaf and grass species tested at 4 kg/ha after 16 days and lethality after 21 days [207].

**Figure 7:**
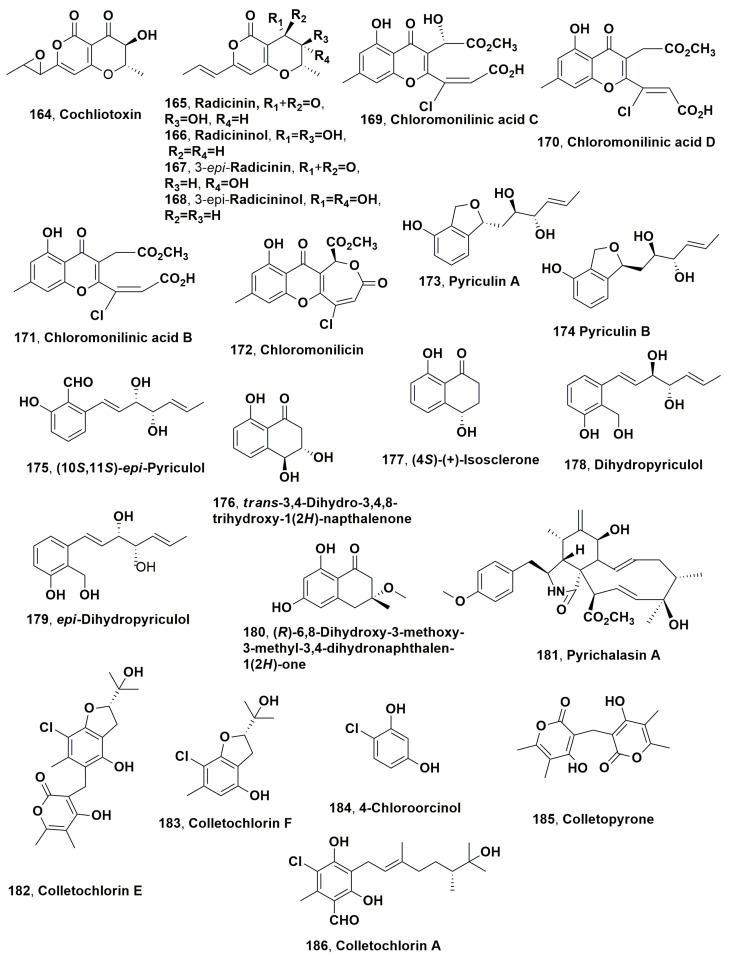
Metabolites produced by *Cochliobolus australiensis* (**164**–**171**), *Ascochyta sonchi* (**172**), *Perycularia grisea* (**173**–**182**) and *Colletotrichum higginsianum* (**183**–**186**).

Cochliotoxin, radicinin, radicinol and their 3-epimers (**164**–**168**, Figure 7) were isolated from *Cochliobolus australiensis*, a foliar fungal pathogen proposed for the biocontrol of buffelgrass (*Pennisetum ciliare* or *Cenchrus ciliaris*). This weed is a perennial grass which is a highly invasive species in the Sonoran Desert of Southern Arizona. All the compound isolated were assayed at a concentration of 2.5–5.0 × 10^−3^ M by coleoptile elongation test and by leaf puncture bioassay on buffelgrass and two nontarget native grasses such as tanglehead (*Heteropogon contortus*) and Arizona cottontop (*Digitaria californica*). Cochliotoxin (**164**) showed strong phytotoxicity, radicinin and 3-*epi*-radicinin (**165** and **167**) also exhibited phytotoxic activity, while radicinol and 3-epi-radicinol (**166** and **168**) were not toxic. All compounds were more active in leaf puncture bioassays on buffelgrass than on the nontarget grass [208]. From the organic extract of the same fungus, grown on different culture mediums were successively isolated as chloromonilinic acids C and D (**169** and **170**, Figure 7), which are two new tetrasubstituted 3-chromanonacrylic acids, together with chloromonilinic acid B (**171**, Figure 7). All three chloromonilinic acids tested 5 × 10^−3^ M were toxic to buffelgrass in a seedling elongation bioassay, with significantly delayed germination and dramatically reduced radicle growth [209]. Chloromonilinic acid B was firstly isolated together with its bromine analogue, chloromonilinic acid A and chloromonilicin (**172**, Figure 7) from the cherry rot fungus *Monilinia fructicola* [210]. Compound **172** inhibited the growth of *M. fructicola*, while chloromonilinic acids A and B were inactive. Chloromonilicin was previously isolated from *A. sonchi*, another fungus proposed for the biocontrol of *S. arvensis* [211].

Pyriculins A and B (**173** and **174**, Figure 7), two monosubstituted hex-4-ene-2,3-diols, were isolated together with (10*S*,11*S*)-(−)-*epi*-pyriculol, *trans*-3,4-dihydro-3,4,8-trihydroxy-1(2*H*)-napthalenone and (4*S*)-(+)-isosclerone (**175–177**, Figure 7) from *Pyricularia grisea* another foliar pathogen of buffelgrass (*C. ciliaris*) in North America proposed for the biocontrol of this very dangerous weed [212]. All the compounds isolated were bioassayed at 5 × 10^−3^ M in a buffelgrass coleoptile and radicle elongation tests. (10*S*,11*S*)-(−)-*epi*-Pyriculol (**175**) exhibited the strongest phytotoxic activity. Seed germination was significantly reduced with respect to the control, and radicles failed to elongate. All five compounds (**173**–**175**) delayed germination, but only (10*S*,11*S*)-(−)-*epi*-pyriculol prevented radicle development of buffelgrass seedlings. Compound **175** had no effect on coleoptile elongation, while the other four compounds markedly induced the increase in coleoptile development relative to the control [212]. Successively from the organic extract of the same culture filtrates were isolated Dihydropyriculol, *epi*-dihydropyriculol, (*R*)-6,8-dihydroxy-3-methoxy-3-methyl-3,4-dihydronaphthalen-1(2*H*)-one and (*R*)-mevalonolactone (4) (**178**–**180**, Figure 7 and **161**, Figure 6) [213] were successively isolated from the organic extract of the same culture filtrates. All the compounds isolate (161 and **178**–**180**) were bioassayed at 5 × 10^–3^ M in a buffelgrass coleoptile and radicle elongation test and no toxicity was detected. Oppositely, compounds **178** and **180** showed a significant stimulating effect of radical elongation. Furthermore, the difference in the induction of growth stimulation observed between compound **178** epimer **179** highlights the relationship between absolute configuration and biological activity of these fungal metabolites as well as in general for all naturally occurring compounds [213].

Pyrichalasin H (**181**, Figure 7) was successively isolated from *P. grisea* but obtained from infected leaves of *Brachiaria eruciformis*, locally known as “signal grass”, which is a common weed in Mississippi, USA. It was tested for germination and growth on *Lactuca sativa* (lettuce; dicot) and *Agrostis stolonife*ra (bentgrass; monocot) seeds in 24-well plates. Pyrichalasin H (**181**) inhibited germination of the monocot at 330 µM, whereas it was lesser toxic against dicot. Compound **181** induced the growth reduction in the monocot duckweed (*Lemna pausicostata*) with IC_50_ value of 150 µM [214].

**Figure 8:**
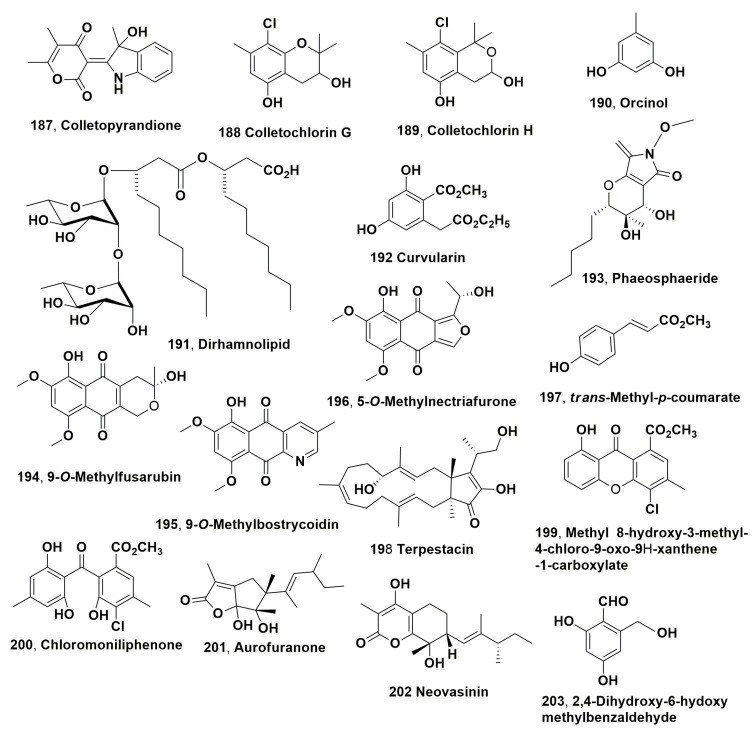
Metabolites produced by *Colletotrichum higginsianum* (**187**–**190**), *Colletotrichum gloeosporioides* (**191**), *Paraphoma* sp. (**192** and **193**), *Rutstroemia capillus-albis* (**194**–**198**), *Alternaria sonchi* (**199** and **200**) and *Ascochyta araujiae* (**201**–**203**).

*Colletotrichum* species caused plant anthracnose diseases, whose symptoms include necrotic spots on leaves, stems, flowers and fruits, although sometime red rot, crown and stem rot, seedling blight and brown blotch were also reported [215]. *Colletotrichum higginsianum*, which belongs to *Colletotrichum destructivum* species complex [216], causes anthracnose leaf spot disease on many cultivated forms of Brassica. Colletochlorins E and F, which are, respectively, a tetrasubstituted pyran-2-one and a dihydrobenzofuran, (**182** and **183**, Figure 7), were isolated together with 4-chloroorcinol, colletopyrone and colletochlorin A, (**184**–**186**; Figure 7) from the culture filtrates of the fungus *C. higginsianum* [217]. When assayed by leaf puncture on *S arvensis* and tomato leaves at 2 μg/μL applying a 20 μL/droplet, compound **184** induced a quite large necrosis (>1 cm), whereas 4-chloroorcinol (**184**) showed the strongest phytotoxicity. Similar results were observed testing compound **183** on *Lemna minor* and *Phelipanche ramosa* seed germination while colletochlorin E, colletopyrone and colletochlorin A (**182**, **185** and **186**) were less or modestly active, respectively [216]. Colletopyrandione and colletochlorins G and H (**187**–**189**, Figure 8) were also isolated from the organic extract of the culture filtrates of the same fungus [218]. Compound **187**, tested at 2 μg/mL applying a 20 μL droplet by the puncture assay on different plant species, such as *S. arvensis*, *H. annuus*, *Convolvulus arvensis* and *Ambrosia artemisiifolia*, induced small but clear necrosis only on *S. arvensis* leaves, and smaller lesion on sunflower. The compounds **188** and **189** were not tested because of the very low amount available [218].

Colletochlorin A, orcinol and tyrosol (**186**, and **190** Figure 8, and **160**) were isolated *Colletotrichum gloeosporioides*, a fungal pathogen proposed to biocontrol ragweed *A. artemisiifolia* L.). This weed is responsible for serious allergies harmful to humans. All the compounds isolated (**160**, **186** and **190**) were tested on *A. artemisiifolia* by leaf puncture, and colletochlorin A (**186**) induced the fastest appearance of large necrosis, while orcinol (**190)** was not toxic. Colletochlorin A, assayed by uptake on *Ambrosia* plantlets, caused the wilting of the plants and the appearance of large leaf necrosis, while the stem appeared only slightly damaged. In the same assay, orcinol was weakly active and tyrosol was not toxic. Colletochlorin A caused clear frond browning on *Lemna minor*, with a total reduction in chlorophyll content of around 70%; the other two compounds were completely inactive [219]. The first asymmetric and total synthesis colletochlorin A (**186**) as well as that of colletorin A was also realized to have amount of both fungal metabolites to further characterize their biological properties [220].

Dirhamnolipid (Rha-Rha-C10-C10) (**191**, Figure 8) was isolated from *C. gloeosporioides* obtained BWH-1 from anthracnose disease-infected Bawanghua (*Hylocereus undatus*) in China [221]. Some varieties of this plant are cultivated for their fruits, while some other varieties were growth for their flowers. Both dried flowers and fruits are used in folk Chinese medicine [222]. Dirhamnolipid showed broad herbicidal activity against eight weed species with IC_50_ values ranging from 28.91 to 217.71 mg/L and no toxicity on *Oryza sativa* [221].

Curvulin and phaeosphaeride (**192** and **193**, Figure 8) were isolated from A *Paraphoma* sp., which was recognised as a pathogen of *C. arvense*. Compounds **192** and **193** were tested on wounded leaf segments of *C. arvense* and *E. repens*, at concentrations ≥840 and 67 and 84 mM, respectively, causing necrotic lesions on leaves within 24 h post-treatment. Compound **192** induced weak phytotoxic activity on *Aamaranthus spinosus* and *Portulaca oleraceae* [223].

Additionally, 9-*O*-Methylfusarubin, 9-*O*-methylbostrycoidin, 5-*O*-methylnectriafurone, *trans*-methyl-*p*-coumarate and terpestacin (**194**–**198**, Figure 8) were isolated from the solid of *Rutstroemia capillus-albis*, which was recognized as the agent responsible of the so-called ‘bleach-blonde syndrome’ on the invasive annual grass weed *Bromus tectorum* (cheatgrass) in Western North America [224]. Compounds **195** and **199** in a juvenile plant immersion bioassay at 10^−4^ M showed strong phytotoxicity, indulging wilting and plant death within 10 days. The other metabolites showed a lesser extended toxicity [224].

Methyl 8-Hydroxy-3-methyl-4-chloro-9-oxo-9*H*-xanthene-1-carboxylate and 5-chloromoniliphenone (**199** and **200**, Figure 8) were isolated together with another eleven closely related metabolites from *A. sonchi* proposed for the biocontrol of perennial sowthistle (*S. arvensis*). The other metabolites were identified as 4-chloropinselin, methyl 3,8-dihydroxy-6-methyl-4-chloro-9-oxo-9*H*-xanthene-1-carboxylate, pinselin, methyl 3,8-dihydroxy-6-methyl-9-oxo-9*H*-xanthene-1-carboxylate, methyl 8-hydroxy-6-methyl-9-oxo-9*H*-xanthene-1-carboxylate, chloromoncilin, moniliphenone, chloromonilinic acids B, C and D and α,β-diversolonic esters. All compounds were tested at concentrations of 2 mg/mL on leaf disks/segments of perennial sowthistle (*S. arvensis*) and couch grass (*E. repens*), showing weak phytotoxicity and inducing lesions up to 2.5 mm in diameter/length [225].

Araufuranone (**201**, Figure 8) was isolated together with neovasinin and 2,4-dihydroxy-6-hydoxymethylbenzaldehyde **(202** and **203**, Figure 8) from *Ascochyta araujiae*, collected from the infected leaves of white blade flower plant (*Araujia hortorum*) [226]. *A. hotorum* is a perennial vining plant species native to South America. It was introduced into many countries for ornamental and medicinal purposes as well as for its edible fruits, but it has become highly invasive, generating severe environmental problems. Assayed by a puncture, on six weeds such as *Calamintha* sp., *Cyperus* sp., *Convolvulus arvensis* L., *Diplotaxis* sp., *Heliotropium europeum* L. and *Sonchus* sp. at 0.8 mg/mL, araufuranone (**201**) showed a weak toxicity on the leaves of *Diplotaxis* sp. and *Sonchus* sp.; the other two metabolites were less toxic. Compounds **202** and **203,** when assayed on cress, caused partially inhibition of rootlet elongation [226].

## 4. Fungal Phytotoxins to Biocontrol Parasitic Plants

Among the parasitic plants, witchweeds (*Striga* spp.) and broomrapes (*Orobanche* spp.) are the two most devastating species causing serious losses on several cereal and leguminous crops, respectively [227]. These parasitic plants are able to produce tiny but large numbers of seeds with prolonged viability and special germination requirements. Strigol, which was isolated from cotton, was discovered as the first very efficacious natural *Striga* germination stimulant starting from 10^−15^ M [228,229]. Successively, dihydrosorgoleone was isolated from sorghum as a stigolactone activity-like compound [230]. The chemical, biological, synthetic and biosynthetic aspects of strigolactones including analogues and derivatives were extensively investigated, and the results were reported in 2516 articles and 525 reviews from the SCiFinder research.

The main aims to control parasitic plants is to drastically reduced their soil seed bank. Two strategies based on the use of natural compounds and seed germination inhibition were proposed to biocontrol parasitic plants: (a) the use of fungal and plant metabolites able to inhibit the seed germination inducing their necrosis; (b) the use of natural compounds able to induce the seed germination in the absence of the host plant causing their aborption; this last strategy is the so-called “suicidal germination” [12,230].

This section chronologically describes, except the cases of treating the same argument, the source, structure and biological activity of the fungal metabolites which showed potential herbicidal activity to biocontrol parasitic plants and in some cases other interesting biological activities.

**Figure 9:**
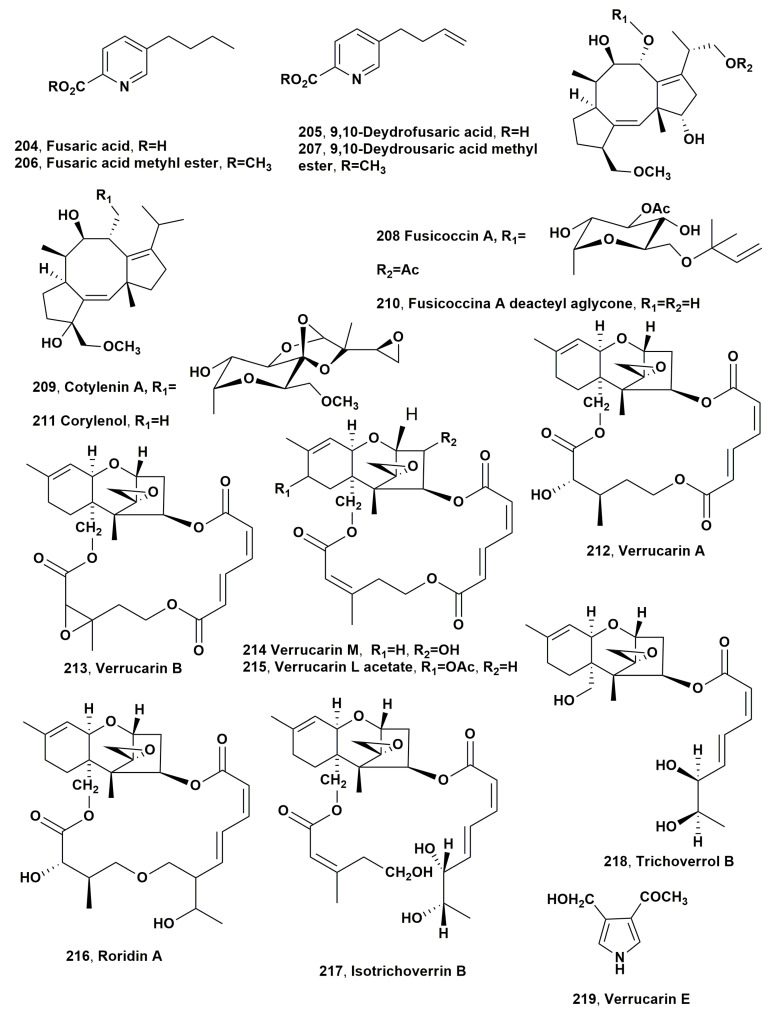
Metabolites produced by *Fusarium nygamai* (**204**–**207**), *Phomopsis amygdali* (**208** and **210**), *Cladosporium* sp. (**209** and **211**) *Myrothecium verrucaria* (**212**–**219**).

Fusaric, 9,10-dehydrofusaric acids (**204** and **205**, Figure 9) and their corresponding methyl esters (**206** and **207**, Figure 9) were isolated from the culture filtrates of *Fusarium nygamai* proposed for the biocontrol of *Striga hermonthica* [231]. *S. hermonthica*, commonly called witchweed, is a parasite weed which causes serious losses in many important cereal crops, such as sorghum, corn, millet, rice and sugarcane [232]. When assayed on tomato leaves and seedlings at 2.7 × 10^−3^ and 2 × 10^−4^ M, respectively, compound **204** and **205** and their methyl esters (**206** and **207**) induced wide chlorosis, which turn into necrosis, as well as in a strong inhibition of root elongation, respectively [230]. Additionally, *Fusarium verticilloides*, isolated from the tubercles of the parasitic weed *Orobanche cumana* in Israel, was able to produce phytotoxins. The fungus tested against *O.cumana*, *Orobanche crenata*, *Pelipanche aegyptiaca* and *Pelipanche ramosa* showing strong toxicity against *P. aegy*ptiaca, *P. ramosa* and *O. cumana*. Its culture filtrates and the corresponding organic extract induced complete mortality of *O. cumana* and *P. aegyptiaca* seedlings in vitro. The toxin was isolated identified as fusaric acid (**205**) by transformation of the parent compound in the corresponding methyl ester [233].

For the control of the same parasitic plant *S. hermontica* as well clover broomrape, *Orobanche minor* were proposed cotylenins (CNs) and fusicoccins (FCs). Cotylenins and fusiccocins are glucosylated diterpenes sharing the same 5:8:5 carbotrycilcyc ring system. Fusicoccin A (FC, **208**, Figure 9) was isolated as the main phytotoxin produced by *Phompsis amygdali* (syn. of *Fusicoccum amygdali*), the causal agent of a devastating disease of almond and peach trees. The surprising story of this phytotoxin, which was started in 1964 and continues today as a potential anticancer drug with more than 1680 citations, was recently reviewed [234]. All the cotyleninns were obtained from culture broth of *Cladosporium* sp. 501-7W [235,236]. Fusicoccin A (FC) and cotylenin A (CN-A) and their aglycones (fucicoccin deactyl aglycone and cotylenol, FC-A and CL) (**208**, **209**, **210** and **211**, Figure 9) at 10^−4^ M strongly induced *Striga* seed germination (60–86% germination), while any or negligible germination was observed in the same seeds incubated in distilled water, and strigol at 10^−8^ M induced higher germination (85–95%). The stimulants activities decreased in the order of strigol, CL, FC, CN-A and FC-A. Under the same experimental conditions, *O. minor* was more sensitive than *S. hermonthica* to all germination stimulants. In fact, strigol at 10^−10^ M induced >80% germination, while CN-A, CL, FC, and FC-A and at 10^−4^ M induced high germination (80–91%). At 10^−5^ M, CL and FC-A showed high activity (∼80% germination), while CN-A and FC induced poor (45%) and moderate (56%) activity, respectively [237]. A SAR study was carried out testing 24 natural analogues and hemisynthetic derivatives of fusicoccin (FC) and cotylenol, assaying their ability to stimulate the seed germination of *P. ramosa*. The natural analogues and derivatives of fusicoccin used were as follows: (a) the glucosides, 8-keto-triacetylFC, 19-deoxydideacetylFC, dideactylFC, 19-deoxy-19-fluorodideactylFC, 19-monoacetyldideacetylFC, 19-deoxy-3α-hydroxydideacetylFC, 3α-hydroxydideacetylFC, 12-monoaceyldideacetylFC, 16-*O*-demethyl-19-deoxydideacetyl-3-*epi*FC, de-*t*-pentenyl-16-*O*-demethyl-19-deoxydideacetylFC, perhydroFC, FC-pseudoacetonide, 8-keto-de-*t*-penyltetracetylFC, two isomers of de-*t*-pentenyletracetyl FC, 16-*O*-demethyl-de-*t*-pentenyl-19-deoxydideacetyl FC; among the aglycones are: (b) deacetylaglyconeFC, aglyconeFC, cotylenol, 8,9-isopropylidenedeacetylagliconeFC, 8,9-isopropylidene-19-deoxydeacetylaglycongeFC, 8,9-isopropylidene-12-keto-19-deocy deacetylaglyconeFC, 8,9-isopropylidene-12-*epi*-19-deoxydeacetylaglyconeFC, 8,9-isopropylidene-19-trityldeacetylaglyconeFC and an isomer of deacetylaglyconeFC. The results obtained showed that 8,9-isopropylidene of the corresponding FC deacetylaglycone and the dideacetylFC were the most active FC derivatives. In both groups of glucosides and aglycones (including cotylenol), the most important structural feature to impart activity appears to be the presence of the primary hydroxy group at C-19. The functionalities and the conformation of the carbotricyclic ring system also play a significant role. Thus, the dideacetyl derivative of FC, which is obtained by easy hydrolysis of the toxin (**209**) and could be prepared in high yield, had a potential for its practical application as a stimulant of *P. ramosa* to its biocontrol based on ‘‘suicidal germination’’ [238]. Successively, considering these results and the structural relation between FC-A (**209**) and ophiobolin A (**66**) and that the stimulation of seed germination is species-dependent, the two toxins and seven of the above cited FC derivatives were assayed at the concentration range of 10^−4^–10^−7^ M on seed germination of different *Orobanche* species such as *P. aegyptiaca*, *P. ramosa*, *O. crenata*, *O. cumana*, *O. densiflora*, *O. fetida*, *O. gracilis*, *O. hederae* and *O. min*or. Among all the compound tested, ophiobolin A and the hexacetyl and pentacetyl isomers of 16-*O*-demethyl-de-*t*-pentenylfusicoccin showed the highest stimulatory effect, while the other fusicoccin derivatives appeared to be practically inactive. The most sensitive species appeared to be *P. aegyptica*, *O. cumana*, *O. minor* and to a lesser extent, *P. ramosa* [239].

**Figure 10:**
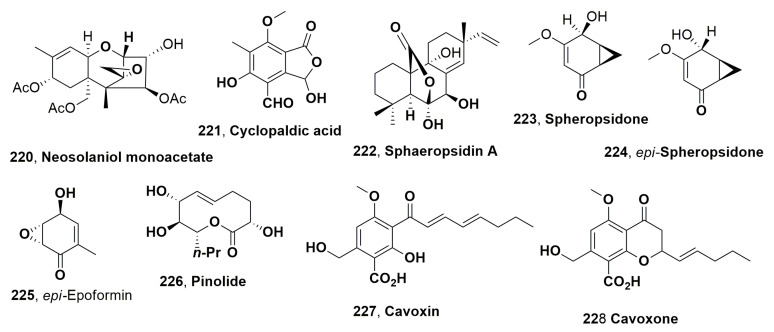
Metabolites produced *by Fusarium co*mpactum (**220**), *Seiridium cupressi* (**221**) *Diplodia cupressi* (**222**–**225**), *Dydimella pinodes* (**226**), *Phoma cava* (**227** and **228**).

Seven macrocyclic trichothecenes, namely verrucarins A, B, M and L acetate; roridin A and isotrichoverrin B; and trichoverrol B and verrucarin E (**212**–**219**, Figure 9) were isolated from *Myrothecium verrucaria* obtained from diseased *P. ramosa* collected in southern Italy. Compound **219**, which is a disubstituted pyrrole, is the main metabolite, *Fusarium compactum* was also isolated from the same infected plant and produced as main metabolites as the trichothecene neosoloaniol monoacetate (**220**, Figure 10). Trichotechenes are well-known sesquiterpenoid mycotoxins produced by different fungi which possess high toxicity for humans and animals [240]. All the compounds isolated were tested on seed of *P. ramosa* according to previously optimized methods [241]. Except for verrucarin E (**219**), which is not a trichothecene and was inactive, all the metabolites **212**–**218** and **220** when assayed at 100 µM totally inhibited the seeds stimulated germination and were still highly active up to 10 µM, causing total inhibition of seed germination, except for isotrichoverrin B and trichoverrol B (**217** and **218**), which were slightly less toxic and almost inactive, respectively [242].

Neosolaniol monoacetate (**220**) and roridin A (**216**) appeared strongly active at 1 µM, causing the total inhibition of seed germination. Verrucarins A, B and L acetate (**212**, **213** and **215**) still showed a reduced activity inducing more than 50% inhibition of germination. Being that active metabolites are considered mycotoxin, their zootoxic activity was also investigated. All the metabolites in the assay on brine shrimps tested at 100 µM caused 100% of larvae mortality with the exception of inactive verrucarin E (**219**) [242]. It is interesting to observe that the phytotoxicity against *P. ramosa* seeds and zootoxicity to larvae seem unrelated. In fact, at 1 µM there were differences in toxicity, with neosolaniol monoacetate and roridin A being more phytotoxic, and verrucarin L acetate and verrucarin M being more zootoxic. Thus, these results could suggest the use of both compounds **216** and **220** as non-toxic bioherbicides as used at the low concentration of 1 mM [242].

The effect on seed germination and radicle growth of *O. crenata*, *O. cumana*, *O. minor*, and *P. ramosa*, of fungal metabolites, belonging to different classes of natural compounds on broomrape seed germination and radicle development, was determined in assays in vitro. The metabolites tested are cyclopaldic acid (**221**, Figure 10), a pentasubstituted benzofuranone, sphaeropsidin A, a pimarane diterpene, sphaeropsidone and *epi*-sphaeropsidone and *epi*-epoformin (**222**–**225**, Figure 10), phytotoxic cyclohexeneepoxides produced, respectively, by *Seiridium cupressi*, the causal agent of canker of Italian cypress (*Cupressus sempervirens* L.) and *Diplodia quercivora* an oak pathogen [243]. In addition, there were also assayed some already reported nonenolides such as pinolidoxin, herbarumin II, 2-*epi*-herbarumin II, (**85**, **87** and **89**) and pinolide (**226**, Figure 10), produced by *D. pinodes*, the responsibility of pea-anthracnose [150], cavoxin and cavoxone (**227** and **228**, Figure 10), a pentasubstituted benzoic acid and the corresponding chromanone, produced by *Phoma cava*, isolated from infected chestnut [244], the already cited chenopodolin (**38**) and chenopodolan C (**41**), and 6-hydroxymellein (**42**), phytotoxins produced by *P. chenopodicola* proposed for the biocontrol of *C. album* [9,95] and by *P. semeni*perda proposed for the biocontrol of *Annual grasses* [175]. Among all the metabolites assayed, *epi*-sphaeropsidone and cyclopaldic acid induced broomrape germination in a species-specific manner. Furthermore, *epi*-epoformin, sphaeropsidin A and cytochalasans inhibited germination of GR24-treated broomrape seeds, while sphaeropsidin A, cycloheheneepoxides and cytochalasans always inhibited the growth of broomrape radicle. Finally, broomrape radicles treated with *epi*-sphaeropsidone, cytochalasins or sphaeropsidinA, respectively, developed a layer of papillae or became necrotic [245]. On the basis of these results, a more in-depth investigation showed that sphaeropsidine and *epi*-sphaeropsidone (**223** and **224**) were able to induce haustorium development in radicles of the parasitic weeds *S. hermonthica*, *O. crenata* and *O. cumana*. A SAR studies was carried out using compound **223** and **224,** their chlorinated analogues, eight derivatives of sphaeropsidone and three of its 5-epimer (**224**). From compound **225,** the acetyl, the 5-azidopentanoyl and *p*-bromobenzoyl esters, an aromatized triacetyl derivative, the corresponding quinone and hydroquinone and bromohydrin, and finally the dihydroxy derivative obtained for reductive opening of the epoxide group were prepared. From compound **225** the acetyl and 5-azidopentanoyl esters and the corresponding hyroquinone were prepared. All the natural and hemisynthetic derivatives were tested on *O. cumana*, *O. crenata* and *S. hermonthica* to find a molecular specificity model required for haustorium induction. The results obtained showed that the haustorium-inducing activity is due to the possibility of converting the natural sphaeropsidones and hemisynthetic derivatives in the corresponding 3-methoxyquinone. Additionally, the absolute configuration at C-5 played a role in the induction of such an activity [246].

## 5. Commercially Available Bioherbicides

Despite the extensive work performed on fungal metabolites with potential practical application as bioherbicide, no article and/or patent report about a product based on a pure or a mixture of the bioactive metabolites was described in the Section 3 and Section 4. This lack of application is essentially due to two limiting factors including the production of the amount of the bioactive metabolite at industrial level by fermentation or the developing of an ecofriendly and convenient simple enantioselective synthesis, as it was too difficult to develop an efficacy formulation for application in the field. Sometimes, another limitation could be their specific phytotoxicity. On the contrary, several products based on the formulation of fungal pathogens for weeds or parasite plants are commercialized since some years as reported in Table 1. Among all the bioherbicides reported in Table 1, to which is associated a commercial name, there are products formulated with fungal belonging to a well-known phytotoxin producer genera such as *Colletortichum*, *Puccinia*, *Chondostereum* and *Sclerotium*. Only one is formulated using a bacteria (*Xanthomonas campetris* sbsp. *poae*), and another one for the first time using a virus (TOBAMOVIRUS). The commercial bioerbicides are used to biocontrol some very dangerous weeds such as *Ascochyta virginica*, *Morrenia odorata*, *Malva pusilla*, *Acacia* spp. *Cyperus esculentus*, *Cuscuta* trees, *Poa annua*, *Solanum viarum* and *Traxonum officinale*. These weeds are very dangerous for cereals cultures (rice and soybean), *Citrus* groves, tree, forests, golf courses, natural environments and other different agrarian cultures [247,248].

Among commercial bioherbicides reported in Table 1 there is Collego, which is a powdery formulation containing 15% of *Colletorichum gliosporioides* f. sp. *aeschynomene* spores. This formulate is able to infect leaves, petioles and stems as well as seeds and seedlings. of *Aeschynomene virginica, Aeschynomene indica and Sesbania exaltata*. Symptoms are visible 7–10 days after application, allowing for 90–100% control. De Vine is sold as a liquid suspension containing 6 × 10^5^ chlamydospores/mL of *Phytophthora palmivora* f. sp. *aeschinomene* to be applied on the surface of the soil. The suspension causes necrosis of stems and death of plants 1–6 weeks from application depending on the age of the *Morrenia odorata* plants. BIOMAL is a wettable powder of *Colletotrichum gleosporioides* f. sp. *malvae*, which is a fungus used to control *Malva pusilla*. It must be kept cold, and the spores require a certain degree of humidity to germinate and penetrate the mallow tissues. It has never had real commercial success because it requires about 20 h of wetting, is difficult to reach in natural conditions, for infection to develop and the disease to appear. Stompout, which is formulated using *Cylindrobasidium leave* spores in South Africa, accelerates the decomposition of stumps and roots of *Acaia* spp. in natural environments. Dr. Biosedge, which is formulate in the Japan using *Puccinia canaliculata* spores, inhibits the reproductive process and seed germination in yellow nutsedge (*Cyperus esculentus* L.) when infested with different agrarian coltures.Myco.Tech was developed in the USA formulating *Chondostereum purpureum* spores. This mycohercide causes stump decay and prevents resprouting of some shrubs such as alder (*Alnus rubra*), pado (*Prunus serotina*) birch (*Betula papyrifera*) and poplar (*Populous* sp.), and is applied in forested areas. Camperico is a bioerbicide developed in Japan formulating the bacterium *Xanthomonas campetstris* subsp. *poae* for the control of the invasive weed *Poa annua*. The bioherbicide suppresses growth and causes black rot disease of weeds in golf courses. SoviNix is formulated as a concentrated liquid or a wettable powder containing tobacco mild green mosaic virus. The bioherbicide triggers a hypersensitive response in *Solanum viarum* and causes necrotic local lesions. It was used to control the invasive weed in perennial grass pastures. Sarritor is a formulate containing *Sclerotium minor*, isolated from lettuce, which absorbs plant tissue and is used to control dandelion (*Taraxacum officinale*) and other broadleaf weeds in turfgrass.

## 6. Application Methods of Bioherbicides

Two different methods are used to apply the bioherbicides reported in Section 5 and are below briefly described.

### 6.1. Liquid Formulations

Post-emergency control with pathogens that essentially cause damage to leaves and stems.

### 6.2. Solid Formulations

Pathogens that induce infection at or below the soil surface.

## 7. Future Perspectives

Future prospects envisage different strategies for improving the efficacy of bioherbicides. The biological processes involve the identification of new targets, new agents, the multiple use of different pathogens and the simultaneous application of phytotoxins and pathogens as well as that of insects and pathogens. The technological strategies include the production methods, the increasing virulence of pathogens and precision agriculture. Integrated pest management involves the use of chemical products and the weed defense suppressants. Genetic methods involve the reduction in pesticide resistance, the development of biomarkers and the assessment of the environmental risks.

## 8. Conclusions

Weeds including parasite plants represent one of the most serious constraints in agrarian and pasture production, in reforestation practices in wood-producing countries, and in protection of forest and ornamental heritage as well as historic garden, archeological ruins and monuments. Severe problems are also generated in important infrastructures such as railways and highways because these plants can represent serious risks for the performance of regular service and cause serious accidents. These infestations determine heavy economic losses and require the use of efficacy methods to combat weeds and parasitic plants diffusion. In the past, agronomic and agrarian methods were used, but because of their low efficacy, these traditional methods were overcome in recent decades by the massive and extensive use of synthetic herbicides, which have generated the large problem of environmental pollution with a consequent contribution to climate changes, and due also to the repetition of treatment and the subsequent resistance developed by weedy plants. Severe risks for human and animal health were frequently recognized as being due to their transmission along the food chain. Thus, peoples and politicians inquire as to the development of alternative methods based on natural substance with herbicidal efficacy, selectivity, and ecofriendly properties. This review reports a complete overview of the fungal metabolites with herbicidal activity, reporting in details their source, the chemical properties, the biological activity and their potential practical application, while in some cases the results of structure-activity relationships studies were also discussed. All the results described for the biocontrol of weeds and parasite plants are summarized in Table 2 and Table 3, respectively. However, for the most promising compounds found in the research, work should evaluate the eco- and human toxicological profile and develop their large production at an industrial level by fermentation or a total convenient and ecofriendly synthesis.

## Figures and Tables

**Table 1:** Bioherbicides commercially available.

Product	Company	Microorganism Formulated	Weed ^1^	Colture ^2^
Collego	TUCO, Amarillo, TX, USA	*Colletotrichum gleosporioides* f. sp. *aeschynomene*	*Aeschynomene virginica, Aeschynomene indica and* *Sesbania exaltata*	Rice, soybean
De Vine	Abott Laboratories, Chicago, IL, USA	*Phytophthora palmivora* f. sp. *aeschinomene*	*Morrenia odorata*	*Citrus* groves
BIOMAL	Novartis Biomedical research, Dorval, QC, Canada	*Colletotrichum gleosporioides* f. sp. *malvae*	*Malva pusilla*	Different
Stompout	CAB International, Wallingford, UK	*Cylindrobasidium leave*	*Acacia* spp.	Natural enviroments
Dr. Biosedge	Bicosis (Adavanced Biocontrol System) Co., Ltd., Geochang-gun Gyeongsangnam-Do, Korea	*Puccinia canaliculata*	*Cyperus esculentus*	Different
Myco.Tech	MycoTechnology, Inc., Aurora, CO, USA	*Chondostereum purpureum*	Trees	Forests
Camperico	Japan Tobacco, Inc., Toranomon, Minato-Ku, Tokyo, Japan	*Xanthomonas campestris* subsp. *poae*	*Poa annua*	Golf courses
SolviNix	BIOPRODEX, INC., Gainesville, FL, USA	Tobacco mild green mosaic virus	*Solanum viarum*	Perennial grass pastures
Sarritor	McGill Spin-off Anomera Inc, Montreal, QC, Canada	*Sclerotim minor*	*Traxonum officinale*	Not reported

^1^ Weed biocontrolled. ^2^ Infested agrarian plant.

**Table 2:** Microbial phytotoxins to biocontrol weeds.

Phytotoxin	Weed	Fungus	Biological Activity	References
Tuenazoic acid (**1**)	Johnsongrass (*Sorghum halepense*) L.	*Alternaria alternata* *Alternaria longipes*	Phytotoxic activity on tobacco leaves	[44] [46]
Monocerin (**2**)	“	*Exserohilum turcicum*	Phytotoxic activity on the host plant, tomato and *Cirsium arvenses*	[47]
Altheichin (**3**)	Water hyacinth (*Eichornia crassipes*)	*Alternaria eichorniae* *Alternaria alternata*	Phytotoxic activity on the host plant water hyacinth (*Eichornia crassipes*) and non-host plant Pigment	[48] [69]
Bostrycin (**4**)	“	*Alternaria eichorniae*	Phytotoxc activity on the host plant water hyacinth (*Eichornia crassipes*) and some agrarian and weedy plants	[49]
4-Deoxybostrycin (**5**)	“	“	“	“
Bipolaroxin (**6**)	Bermuda grass (*Cynodon dactylon* L.)	*Bipolaris cynodontis*	Specific activity against the two host plants Bermuda grass and johnson grass	[50]
Dihydrobipolaroxin (**7**)	“	“	No toxicity	“
Cyclo(-L-Pro-L-Tyr-) (**8**)	Knapweed, (*Centaurea maculosa*)	*Alternaria alternata*	Phytotoxic activity against the host knapweed, Antifungal Antibiotic	[51] [53,55] [60]
Cyclo(-L-Pro-L-Phe-) (**9**)	“	“	Phytotoxic activity against the host knapweed	[51]
cyclo(-L-Pro-D-Phe-) (**10**)	“	“	No toxic on host plant	“
cyclo(-Pro-Hle-) (**11**)	“	“	“	“
cyclo(-Pro-Val-) (**12**)	“	“	“	“
cyclo(-Pro-Leu-) (**13**)	“	“	“	“
cyclo(-Pro-Ala-) (**14**)	“	“	“	“
cyclo(-L-Pro-L-Val-) (**15**)	-	*Lysobacter capsici*	Antifungal activity Antibiotic activity	[60]
cyclo(-D-Pro-D-Phe-) (**16**)	“	“	Antifungal activity	“
cyclo(-L-Pro-L-Leu-) (**17**)	“	“	“	“
cyclo(-D-Pro-L-Tyr-) (**18**)	“	“	“	“
Alterlosin I (**19**)	Knapweed (*Centaurea maculosa*)	*Alternaria alternata*	Phytotoxic on knapweed	[68]
Alterlosin II (**20**)	“	“	“	“
Altertoxin III (**21**)	“	“	Phytotoxic on lettuge Mutagenic.	[69] [70]
Viridiol (**22**)	Several spp.	*Gliocladium virens* *Hymenoscyphus* *fraxineus*	Phytotoxic activity on weeds Phytotoxic activity on some forest plant	[71] [74]
Gliotoxin (**23**)	Several spp.	*Gliocladium virens*	Antibiotic, Antifungal	[71,72]
Gliovirin (**24**)	“	“	“	[71,73]
Cyperine (**25**)	Purple nutsedge (*Cyperus rotundus* L.)	*Ascochyta cypericola*	Phytotoxic activity on *Cyperus* spp.	[76]
6-Methylsalicylic acid (**26**)	Pockeweed (*Phytolacca americana* L.)	*Phoma sorghina*	Phytotoxic activity on the host pokeweed and 8 other weeds	[77]
Epoxydon (**27**)	“	“	“	“
Desoxyepoxydon (**28**)	“	“	“	“
Phyllostine (**29**)	“	“	“	“
Epoxydon 6-methylsalicylate ester (**30**)	“	“	“	“
Ascochytine (**31**)	Lambsquarters or ft hen (*Chenopodium album* L.)	*Ascochyta hyalospora*	Phytotoxic activity on the host lambsquarters and 8 other weeds	[84]
Pyrenolide A (**32**)	“	“	“	
Hyalopyrone (**33**)	“	“	“	“
Ascosalitoxin (**34**)	Pea and bean (*Pisum sativum* and *Phaseolus vulgaris*)	*Ascochyta pisi*	Phytotoxic activity on the host plants	[85]
Ascaulitoxin (**35**)	“	*Ascochyta caulina*	Phytotoxic activity on the host lambsquarters	[88]
*trans*-4-Aminoproline (**36**)	“	“	“	[89]
2,4,7-Triamino-5-hydroxyoctandioic acid (**37**)	“	“	“	[90]
Chenopodolin (**38**)	“	*Phoma chenopodiicola*	Phytotoxic activity on *Mercurialis annua*, *Cirsium arvense* and *Setaria viride*	[95]
Chenopodolan A (**39**)	“	“	Phytotoxic activity on *Sonchus oleraceus*, *M. annua* and *C. album*	[96]
Chenopodolan B (**40**)	“	“	“	“
Chenopodolan C (**41**)	“	“	No phytoxicity	“
(*R*)-6-Hydroxymellein (**42**)	“	“	No phytoxicity	“
Chenopodolan D (**43**)	“	“	Phytotoxic activity on *Sonchus arvensis*, *Urtica dioica* and *Parietaria officinalis*	[97]
Chenisocoumarin (**44**)	“	“	No phytotoxic	“
Chenopodolin B (**45**)	“	“	Phytotoxic activity on *S. arvensis*, *U. dioica* and *P. officinalis*	“
Chenopodolan E (**46**)	“	“	Zootoxic acrivity	[98]
Chenopodolan F (**47**)	“	“	Phytotoxic activity on *S. arvensis*	“
Convolvulanic acid A (**48**)	Bindweed (*Corzvolvulus arvensis*)	*Phomopsis convolvulus*	Phytotoxic activity against bindweed (*Corzvolvulus arvensis*)	[99]
Convolvulanic acid B (**49**)	“	“	“	“
Convolvulol (**50**)	“	“	“	“
Convolvulopyrone (**51**)	“	“	“	“
Ergosterol (**52**)	“	“	No toxicity	“
Ergosterol peroxide (**53**)	“	“	“	“
Gigantenone (**54**)	Several grasses as weed cabgrass (*Digitaria* spp.), quackgrass (*Agropyron repens*) and Bermuda grass (*Cynodon dactylon*)	*Drechslera gigantea*	Phytotoxic activity on monocot species	[100]
Phaseolinone (**55**)	“	“	“	“
Petasol (**56**)	“	“	“	“
Sesquiterpenes (**57**)	“	“	Not Toxic	“
Sequiterpene(**58**)	“	“	Phytotoxic activity on dicot species	“
Phomenone (**59**)	“	“	Phytotoxic activity on monocot species	“
Sesquiterpenes (**60**)	“	“	Phytotoxic activity on dicot species	“
Sesquiterpenes (**61**)	“	“	“	“
Sesquiterpenes (**62**)	“	“	Not Toxic	“
Sesquiterpenes (**63**)	“	“	“	“
Sesquiterpenes (**64**)	“	“	“	“
Sesquiterpenes (**65**)	“	“	“	“
Ohpiobolin A (**66**)	“	“	Phytotoxic activity against some monocot grasses and dicot weeds	[105]
6-*epi*-Ophiobolin A (**67**)	“	“	Reduced phytotoxicity against some monocot grasses and dicot weeds	“
3-Anhydro-6-*epi*-ophiobolin A (**68**)	“	“	“	“
Ophiobolin I (**69**)	“	“	No Toxicity	“
ophiobolin E (**70**)	“	“	“	[118]
8-*epi*-Ophiobolin J (**71**)	“	“	“	“
Ophiobolins B (**72**)	“	“	Phytotoxic activity against some weeds	“
Ophiobolin J (**73**)	“	“	Reduce or no toxicity against some weeds	“
Drophiobiolins A (**74**)	“	“	Phytotoxic activity on host, other weed and agrarian plants Cytotoxic	[119]
Drophiobiolins B (**75**)	“	“	“	“
AAL-toxin (**76**)	Jimsonweed (*Datura stramonium*)	*Alternaria alternata*	Phytotoxic activity on jimsonweed	[120]
Alternariol monomethyl ether (**77**)	“	“	No toxicity	“
Depudecin (**78**)	Kuroguwai (*Eleocharis kuroguwai*)	*Nimbya scirpicola* *Alternaria brassicola*	Phytotoxic activity on host weed and some crops Anticancer	[134] [135]
Ferrocinin (**79**)	blackberry (*Rubu*s spp.)	*Colletotrichum gloeosporioides*	Phytotoxic activity on some weeds	[136]
Putaminoxin (**80**)	Annual flaebane (*Erigeron annuus*)	*Phoma putaminum*	Phytotoxic activity on the host *annual fleabane*, some other weeds and cultivated plants	[141]
Putaminoxin B (**81**)	“	“	No toxicity	[142]
Putaminoxin C (**82**)	“	“	Phytotoxic activity on annual dog’s mercury, globe artichoke, tomato.	“
Putaminoxin D (**83**)	“	“	No toxicity	[143]
Putaminoxin E (**84**)	“	“	“	“
Pinolidoxin (**85**)	*Pisum sativum* L.	*Dydimella pinodes*	Phytotoxic activity on some weed and crop plants	[144]
Herbarumin I (**86**)	Prine’s father (*Amaranthus hypochondriacus*)	*Phoma herbarum*	Phytotoxic activity against *Amaranthus hypochondriacus*	[147]
Herbarumin II (**87**)	“	“	“	“
Herbarumin III (**88**)	“	“	“	[148]
2-*epi*-Herbarumin II (**89**)	Pendulus yucca (*Yucca recurvifolici*)	*Paraphaeosphaeria recurvifoliae*	Inhibition of murine tyrosinase	[149]
Stagonolide A (**90**)	*Cirsium arvense* (Canada thisle)	*Stagonospora cirsii*	Phytotoxic activity against the host plant *C. arvense*, and several other weeds and cultivated plants	[151]
Stagonolide B (**91**)	“	“	No phytotoxicity	[152]
Stagonolide C (**92**)	“	“	“	“
Stagonolide D (**93**)	“	“	“	“
Stagonolide E (**94**)	“	“	“	“
Stagonolide F (**95**)	“	“	“	“
Stagonolide G (**96**)	“	“	“	[153]
Stagonolide H (**97**)	“	“	Strong phytotoxicity against the host plants	“
Stagonolide I (**98**)	“	“	Moderate phytotoxicity against the host plants	“
Stagonolide J (**99**)	“	“	No toxicity	[154]
Stagonolide K (**100**)	“	“	Phytotoxicity against *C. arvense* and *S. arvensis*	“
Modiolide A (**101**)	“	“	Moderate phytotoxicity against the host plants	[153]
Dendryol A (**102**)	Kuroguwai (*Eleocharis kuroguwai*)	*Dendryphiella* sp.	Phytotoxic activity against barnyardgrass	[159]
Dendryol B (**103**)	“	“	“	“
Dendryol C (**104**)	“	“	“	“
Dendryol D (**105**)	“	“	“	“
Australifungin (**106**)	No infected weed	*Sporormiella australis*	Phytotoxicity against duckweed (*Lemna pausicostata*)	[160]
Brefeldin (**107**)	*Alternaria zinniae*	*Xanthium occidentale*.	Phytotoxicity against the host plant and other weeds	[161]
α,β-Dehydrocurvularin (**108**)	“	“	“	“
2,4-Dihydro-4-(β-D-ribofuranosyl)-1,2,4(3*H*)-triazol-3-one (**109**)	No infected weed	*Actinomadura* sp.	Phytotoxic activity on several weeds	[163,168]
Hydantocidin (**110**)	“	*Streptomyces hygroscopicus*	“	[165,168]
Naphthopyranone derivatives (**111**)	“	*Guanomyces polythrix*	Phytotoxic activity against *Amaranthus hypochondriacus* and *Echinochloa crusgalli*	[173]
Naphthopyranone derivatives (**112**)	“	“	“	“
Naphthopyranone derivatives (**113**)	“	“	“	“
Naphthopyranone derivatives (**114**)	“	“	“	“
Naphthopyranone derivatives (**115**)	“	“	“	“
Rubrofusarin B (**116**)	“	“	“	“
Emodin (**117**)	“	“	“	“
Citrinin (**118**)	“ hemp dogbane (*Apocynum cannabinum* L.).	“ *Stagonospora apocyni*	“ Toxicity on several weeds	“ [174]
4-Hydroxybenzoic acid methyl ester (**119**)	No infected weed	*Guanomyces polythrix*	Phytotoxic activity against *Amaranthus hypochondriacus* and *Echinochloa crusgalli*	[173]
Cytochalasin Z1 (**120**)	Annual grasses (*Bromus* spp.)	*Pyrenophora semeniperda*	Phytotoxicity on wheat and tomato	[175]
Cytochalasin Z2 (**121**)	“	“	“	“
Cytochalasin Z3 (**122**)	“	“	“	“
Cytochalasins F (**123**)	“	“	“	“
Cytochalsin T (**124**)	“	“	“	“
Deoxaphomin (**125**)	“	“	“	“
Cytochalasins B (**126**)	“	“	“	“
Cytochalasin A (**127**)	*Oleander nerium* L.	*Phoma exigua* var. *heromorpha*	Not tested on weed	[178]
Spirostaphylotrichin A (**128**)	Cheatgrass (*Bromus tectorum*)	*Pyrenophora semeniperda*	Phytotoxicity on cheatgrass and non-host plants	[185]
Spirostaphylotrichin C (**129**)	“	“	“	“
Spirostaphylotrichin D (**130**)	“	“	“	“
Spirostaphylotrichin R (**131**)	“	“	Non-toxic on cheatgrass	“
Spirostaphylotrichin V (**132**)	“	“	Phytotoxicity on cheatgrass	“
Spirostaphylotrichin W (**133**)	“	“	“	“
Triticone E (**134**)	“	“	Non-toxic on cheatgrass	“
Pyrenophoric acid (**135**)	“	“	Phytotoxic activity on cheatgrass	[186]
Pyrenophoric acid B (**136**)	“	“	“	[187]
Pyrenophoric acid C (**137**)	“	“	“	“
Abscisic acid (**138**)	“	“	“	“
Macrocidins A (**139**)	Canada thistle (*Cirsium arvense*)	*Phoma macrostoma*	Phytotoxicity on different weeds	[189]
Macrocidins B (**140**)	“	“	“	“
Ascosonchine (**141**)	Sowthistle (*Socnhus arvensis*)	*Ascochyta sonchi*	Phytotoxicity on several weed and cultivated plants	[190]
Drazepinone (**142**)	Ryegrass (*Lolium perenne*)	*Drechslera siccans*	Phytotoxicity on several weeds	[191]
Phyllostoxin (**143**)	Canada thistle *Cirsium arvense*	*Phyllosticta cirsii*	Phytotoxicity on host plant	[192]
Phyllostin (**144**)	“	“	No toxicity	“
Scytolide (**145**)	Pine-pine gall rust (*Endocronartium harknessii*)	*Scytalidium uredinicola*	Inhibiotion of inhibition of germination of *E. harknessii* spores	[193]
Cinnamicidin (**146**)	Not identified	*Nectria* sp.	Phytotoxicity on several weeds	[194]
Coronatine (**147**)	“	*Pseudomonas syringae*	“	[195]
Jasmonic acid (**148**)	“	Plant hormone and fungal metabolites	“	[196]
Papyracillic acid (**149**)	Quack grass (*Elytrigia repens*)	*Ascochyta agropyrina* var. *nana*	Phtotoxicity against different weeds	[197]
Papyracillic acid methyl acetal (**150**)	“	“	“	“
Agropyrenol (**151**)	“	“	“	[200]
Agropyrenal (**152**)	“	“	“	“
Agropyrenone (**153**)	“	“	Not toxic	“
Phomentrioloxin (**154**)	Saffron twistle (*C. arvense*)	*Phomopsis* sp.	Phytotoxicity on several weeds and some cultivated plants	[202]
Gulypyrone A (**155**)	“	*Dyaporthe gulyae*	No toxicity	[203]
Gulypyrone B (**156**)	“	“	Phytotoxicity on *Helianthus annuus* plantlets	“
Phomentrioloxin B (**157**)	“	“	Phytotoxicity on several weeds and some cultivated plants	“
Phomentrioloxin C (**158**)	“	“	“	“
Anhydromevalonolactone (**159**)	wild poinsettia (*Euphorbia heterophylla*)	*Alternaria euphorbiicola*	Phytotoxicity on host plant and other weed	[204]
Tyrosol (**160**)	“	“	“	“
(*R*)-(–)-Mevalonolactone (**161**)	“	“	“	“
Cycloglycylprolin (**162**)	“	“	Selective toxicity against the host plant	“
Mevalocidin (**163**)	No infected weed	*Coniolariella* sp.	Phytotoxicity against broadleaf and grass species	[206,207]
Cochliotoxin (**164**)	buffelgrass (*Pennisetum ciliare* or *Cenchrus ciliaris*)	*Cochliobolus australiensis*	Phytotoxic activity on the host plant and other two native weeds	[208]
Radicinin (**165**)	“	“	“	“
Radicinol (**166**)	“	“	Not toxic	“
3-*epi*-Radicinin (**167**)	“	“	Phytotoxic activity on the host plant and the other two native weeds	“
3-*epi*-Radicinol (**168**)	“	“	Not toxic	“
Chloromonilinic acid C (**169**)	“	“	Phytotoxicity on the host plant	[209]
Chloromonilinic acid D (**170**)	“	“	“	“
Chloromonilinic acid B (**171**)	“	“	“	“
Chloromonilicin (**172**)	Sowthistle (*Socnhus arvensis*)	*A. sonchi*	No toxicity	[1]
Peryculin A (**173**)	buffelgrass (*Pennisetum ciliare* or *Cenchrus ciliaris*)	*Perycularia grisea*	Phytotoxicity on the host weed	[212]
Peryculin B (**174**)	“	“	“	“
(10*S*,11*S*)-(−)-*epi*-Pyriculol (**175**)	“	“	“	“
*trans*-3,4-Dihydro-3,4,8-trihydroxy-1(2*H*)-napthalenon (**176**)	“	“	“	“
4*S*)-(+)-Isosclerone (**177**)	“	“	“	“
Dihydropyriculol (**178**)	“	“	No toxicity	[213]
*epi*-Dihydropyriculol (**179**)	“	“	“	“
3-Methoxy-6,8-dihydroxy-3-methyl-3,4-dihydroisocoumarin (**180**)	“	“	“	“
Pyrichalasin H (**181**)	Signal grass (*Brachiaria eruciformis*)	“	Phytotoxicity on weeds and cultivated plants	[214]
Colletochlorin E (**182**)	*Brassica* sp.	*Colletetotrichum higginsianum*	Phytotoxicity on weeds, parasitic and cultivated plants	[217]
Colletochlorin F (**183**)	“	“	“	“
4-Chlororcinol (**184**)	“	“	“	“
Colletopryrone (**185**)	“	“	“	“
Colletochlorin A (**186**)	Ragweed (*Ambrosia artemisiifolia*)	“ *Colletotrichum* *gloeosporioides*	“ Phytotoxicity on the host weed	“ [218]
Colletopyrandione (**187**)	Rape (*Brassica* sp.)	*C. higginsianum*	Phytotoxicity on *S. arvensis* and *Helianthus annuus*	[218]
Colletochlorin G (**188**)	“	“	Not tested	“
Colletochlorin H (**189**)	“	“	“	“
Orcinol (**190**)	Ragweed (*A. artemisiifolia*)	*C. gloeosporioides*	Phytotoxicity on the host weed	[219]
Dirhamnolipid (**191**)	Bawanghua (*Hylocereus undatus*)	“	Phytoxicity against different weeds	[221]
Curvulin (**192**)	Saffron thisle (*C. arvense*)	*Paraphoma* sp.	No toxicity	[223]
Phaeosphaeride (**193**)	“	“	“	“
9-*O*-Methylfusarubin (**194**)	Cheatgrass (*Bromus tectorum*)	*Rutstroemia capillus-albis*	Phytotoxicity on the host plant	[224]
9-*O*-Methylbostrycoidin (**195**)	“	“	Weak phytotoxicity on the host plant	“
5-*O*-Methylnectriafurone (**196**)	“	“	“	“
*trans*-Methyl-*p*-coumarate (**197**)	“	“	“	“
Terpestacin (**198**)	“	“	“	“
8-Hydroxy-3-methyl-4-chloro-9-oxo-9H-xanthene-1-carboxylate (**199**)	Canada Thisle (*S. arvensis*)	*Alternaria sonchi*	Phytotoxicity on *S. arvensis* and *E. repens*	[225]
Chloromoniliphenone (**200**)	“	“	“	“
Araufuranone (**201**)	White blade flower (*Araujia hortorum*)	*Ascochyta araujiae*	Phytotoxicity on different weeds	[226]
Neovasinin and (**202**)	“	“	“	“
2,4-Dihydroxy-6-hydoxy Methylbenzaldehyde (**203**)	“	“	“	“

“—Means the same content.

**Table 3:** Microbial phytotoxins to biocontrol parasitic plants.

Phytotoxin	Weed	Fungus	Biological Activity	References
Fusaric acid (**204**)	Purple witchweed (*Striga hermonthica*) *Broomrape* (*Orobanche cumana*)	*Fusarium nygamai* *Fusarium verticilloides*	Phytotoxicity on tomato and inhibition of *Striga hermonthica* seeds germination Phytotoxicity against *P. aegy*ptiaca, *O. ramosa* and *O. cumana*	[231] [232]
9,10-Deydrofusaric acid (**205**)	“	“	“	“
Fusaric acid methyl ester (**206**)	“	“	“	“
9,10-Deydrofusaric methyl ester (**207**)	“	“	“	“
Fusicocin (**208**)	No infected weed	*Phomopsis amygdaly*	Stimulation of seeds germination of S*. hermonthica* and *O. minor*	[237]
Cotylenin A (**209**)	“	*Cladosporium* sp. 501-7W	“	“
Fusicoccin deacetyl aglycone (**210**)	“	*P. amygdaly*	“	“
Cotylenol (**211**)	“	*Cladosporium* sp. 501-7W	“	“
Verrucarin A (**212**)	*Broomrape* (*Pelipanche ramosa*)	*Myrothecium verrucaria*	Inhibition of *P. ramosa* seed germination	[242]
Verrucarin B (**213**)	“	“	“	“
Verrucarin M (**214**)	“	“	“	“
Verrucarin L acetate (**215**)	“	“	“	“
Roridin A (**216**)	“	“	“	“
Isotrichoverrin B (**217**)	“	“	“	“
Trichoverrol B (**218**)	“	“	“	“
Verrucarin E (**219**)	“	“	Not toxic	“
Neosolanial monoactate (**220**)	“	*Fusarium compactum*	Inhibition of *P. ramosa* seed germination	“
Cyclopaldic acid (**221**)	No infected weed	*Diplodia cupressi*	Phytotoxicity on *O. crenata*, *O. cumana*, *O. minor*, and *P. ramosa*	[243]
Sphaeropsidin A (**222**)	“	“	“	“
Sphaeropsidone (**223**)	“	“	“	“
*epi*-Spheropsidone (**224**)	“	“	“	“
*epi*-Epoformin (**225**)	“	“	“	“
Pinolide (**226**)	“	*D. pinodes*	No toxicity	[150]
Cavoxin (**227**)	“	*Phoma cava*	No toxicity	[244]
Cavoxone (**228**)	“			

“—Means the same content.

## Data Availability

The literatures used to prepare this review were found on scifinder.cas.org/scifinder.
